# Myocyte enhancer factor (MEF)-2 plays essential roles in T-cell transformation associated with HTLV-1 infection by stabilizing complex between Tax and CREB

**DOI:** 10.1186/s12977-015-0140-1

**Published:** 2015-02-27

**Authors:** Pooja Jain, Alfonso Lavorgna, Mohit Sehgal, Linlin Gao, Rashida Ginwala, Divya Sagar, Edward W Harhaj, Zafar K Khan

**Affiliations:** Department of Microbiology & Immunology and Drexel Institute for Biotechnology & Virology Research, Drexel University College of Medicine, 3805 Old Easton Road, Doylestown, PA 18902 USA; Department of Oncology, Sidney Kimmel Comprehensive Cancer Center, Johns Hopkins School of Medicine, Baltimore, MD 21287 USA; Graduate Program in Cancer Biology, Sylvester Comprehensive Cancer Center, The University of Miami, Miller School of Medicine, Miami, FL 33136 USA

**Keywords:** HTLV-1, Tax, LTR, Retroviral promoter, Myocyte enhancer factor-2 (MEF-2)

## Abstract

**Background:**

The exact molecular mechanisms regarding HTLV-1 Tax-mediated viral gene expression and CD4 T-cell transformation have yet to be fully delineated. Herein, utilizing virus-infected primary CD4^+^ T cells and the virus-producing cell line, MT-2, we describe the involvement and regulation of Myocyte enhancer factor-2 (specifically MEF-2A) during the course of HTLV-1 infection and associated disease syndrome.

**Results:**

Inhibition of MEF-2 expression by shRNA and its activity by HDAC9 led to reduced viral replication and T-cell transformation in correlation with a heightened expression of MEF-2 in ATL patients. Mechanistically, MEF-2 was recruited to the viral promoter (LTR, long terminal repeat) in the context of chromatin, and constituted Tax/CREB transcriptional complex via direct binding to the HTLV-1 LTR. Furthermore, an increase in MEF-2 expression was observed upon infection in an extent similar to CREB (known Tax-interacting transcription factor), and HATs (p300, CBP, and p/CAF). Confocal imaging confirmed MEF-2 co-localization with Tax and these proteins were also shown to interact by co-immunoprecipitation. MEF-2 stabilization of Tax/CREB complex was confirmed by a novel promoter-binding assay that highlighted the involvement of NFAT (nuclear factor of activated T cells) in this process via Tax-mediated activation of calcineurin (a calcium-dependent serine-threonine phosphatase). MEF-2-integrated signaling pathways (PI3K/Akt, NF-κB, MAPK, JAK/STAT, and TGF-β) were also activated during HTLV-1 infection of primary CD4^+^ T cells, possibly regulating MEF-2 activity.

**Conclusions:**

We demonstrate the involvement of MEF-2 in Tax-mediated LTR activation, viral replication, and T-cell transformation in correlation with its heightened expression in ATL patients through direct binding to DNA within the HTLV-1 LTR.

**Electronic supplementary material:**

The online version of this article (doi:10.1186/s12977-015-0140-1) contains supplementary material, which is available to authorized users.

## Background

HTLV-1 is the etiologic agent of adult T cell leukemia (ATL), a progressive lymphoma, and HTLV-1 associated myelopathy/tropical spastic paraparesis (HAM/TSP) [1-6]. Globally, as many as 20 million people are infected with HTLV-1 [7], yet most remain asymptomatic carriers [8-10]. The molecular mechanisms associated with driving HTLV-1 into a quiescent versus active replication mode are not clearly understood; however, it is well established that expression of the viral transactivator protein, Tax, is required for the efficient viral gene expression [11-13].

Upon entering its target cell (CD4^+^ T cells, primarily CD4^+^CD25^+^ cells), HTLV-1 ssRNA(+) is reverse transcribed and integrated semirandomly into the host genome as a provirus [14]. Proviral gene expression depends on Tax [11-13,15,16] and specific cellular transcription factors (TFs) during both primary infection and viral reactivation from latency [17,18]. Tax is a 40 kDa protein that cannot bind to DNA directly but interacts with specific TFs and coactivators that facilitate its binding to Tax-responsive elements (TRE) on the long terminal repeat (LTR) [19-21]. cAMP response element homologous sequences within TRE provide CRE-binding protein (CREB) docking sites where Tax interacts with the CREB homodimer on the viral LTR [22-24]. Tax/CREB binding to the viral promoter initiates the recruitment of histone acetyl transferases (HATs) including p300, p300/CREB-binding protein (CBP), and p300/CBP associated factor (p/CAF) [25-28], which enables nucleosome disassembly and transcriptional activation [29-32]. The role of TFs other than CREB needs to be delineated in HTLV-1 promoter activation and gene expression. Here, we present investigations on Tax-mediated LTR activation in the context of viral infection using HTLV-infected primary CD4^+^ T cells and the virus-producing T cell line (MT-2) and describe the involvement of myocyte enhancer factor-2 (MEF-2) in this process.

MEF-2 was originally described as a transcriptional regulator in cardiac and skeletal muscle [33-35]. In mammals, there are four MEF-2 genes that give rise to MEF-2A, MEF-2B, MEF-2C, and MEF-2D. At the N-terminus, four isotypes are highly conserved and contain a MADS (MCM1, Agamous, Deficiens and serum response factor) domain whereas the highly variable C-termini contain transcription activation domains (TADs), which recruit coactivators, histone modification enzymes and factors associated with nucleosome disassembly and remodeling to activate gene expression [33-35]. All MEF-2 isoforms exhibit significant amino acid sequence similarity within their DNA binding domain [36]. MEF-2 is expressed at high levels in neurons and lymphocytes, where it serves as a regulator of neuronal and immune cell differentiation and function [37,38]. MEF-2A expression is quite ubiquitous unlike MEF-2C and -2B, which remain somewhat tissue tropic, and MEF-2D is a comparatively weak transcriptional activator [39]. The studies presented herein primarily refer to MEF-2A since most of available reagents (antibodies, plasmids, etc.) are specific to this isoform of MEF-2. It is also true for the majority of published work on this cellular factor.

MEF-2 is necessary for the transcriptional activation of Interleukin 2 (IL-2) during peripheral T cell activation [40]. It plays a crucial role in T-lymphocyte apoptosis by regulating expression of Nuclear receptor subfamily 4, group A, member 1 (NUR77) [41,42]. Within T lymphocytes, MEF-2 activity is subjected to complex levels of regulation. MEF-2 associates with a variety of regulating proteins: p300, p/CAF, Nuclear factor of activated T-cells, cytoplasmic, calcineurin-dependent 2 (NFATC2), Nuclear receptor coactivator 2 (NCOA2), Myogenic differentiation 1 (MYOD), Mitogen-activated protein kinase 7 (MAPK7), Calcineurin binding protein 1 (CABIN1), Class II histone deacetylases (HDAC4, HDAC5, HDAC7, HDAC9) [41,43] and is regulated by MAP kinase cascades and calcium signaling.

Association of Class II histone deacetylases such as HDAC9 results in deacetylation of nucleosomal histones surrounding MEF-2 DNA-binding sites, with subsequent suppression of MEF-2-dependent genes [38]. MAPKs couple MEF-2 to multiple signaling pathways for cell growth and differentiation [44-47]. MAPK14 and MAPK11 phosphorylate and activate MEF-2A and MEF-2C, and MAPK7 is capable of phosphorylating and activating MEF-2A, MEF-2C and MEF-2D [48,49]. MAPK7 and Extracellular signal-regulated kinase 5 (ERK5), itself are phosphorylated and activated by MEKK2 and 3 [50]. In response to MAPK7, MAPK11 and MAPK14, MEF-2 activates the transcription factor Jun oncogene (c-Jun), which participates in regulation of proliferation [38,51,52]. Interestingly, MAP kinases are associated with HTLV-1 pathogenesis [53], and Tax protein has been shown to activate p38 [54]. Since both p38 and ERK5 activate MEF-2 by phosphorylation [44,49,55-60], it is highly likely that activation of these signaling pathways by Tax activate MEF-2. Therefore, we sought to determine if MEF-2 participates in Tax-mediated transactivation of HTLV-1 promoter.

Not much is known about the role of MEF-2 in viral pathogenesis except for the Epstein-Barr virus (EBV) infection. EBV latency has been linked with chromatin remodeling through the recruitment of MEF-2 to the viral promoter [61-63]. Here, utilizing HTLV-infected primary CD4 T cells, we provide the first evidence for the involvement of MEF-2 in Tax-mediated LTR activation, viral replication, and T-cell transformation in correlation with its heightened expression in ATL patients. MEF-2 was also shown to directly bind to DNA within the HTLV-1 LTR to an imperfect site. Molecular mechanisms involved activation and recruitment of MEF-2 on the LTR in the context of chromatin and co-regulation of transcriptional complex involving both Tax and CREB.

## Results

### MEF-2 inhibition reduces Tax-mediated LTR trans-activation, and viral replication

**Figure 1:**
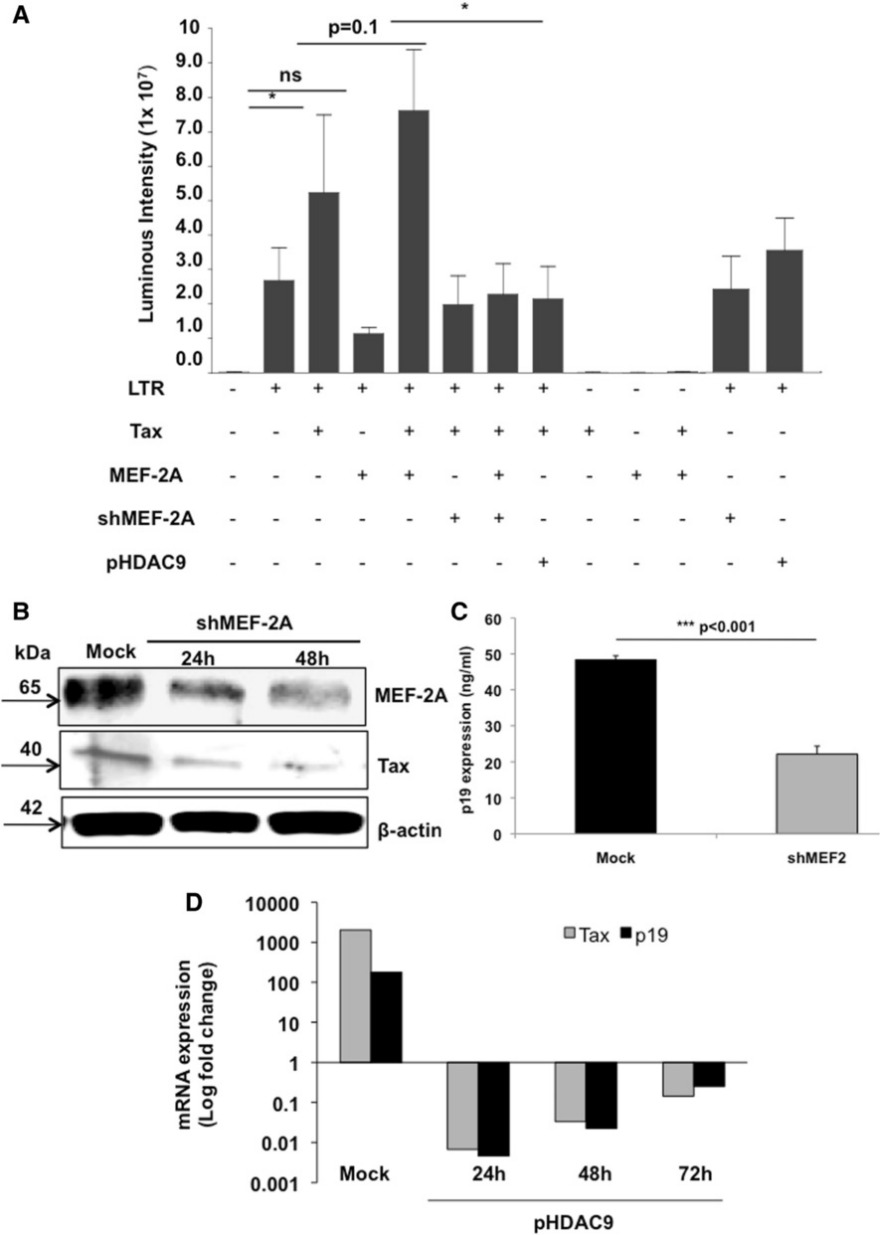
**MEF-2 inhibition reduces HTLV-1 LTR transactivation, Tax expression, and viral replication. (A)** Transient transfection of Jurkat cells with pU3R-luc (HTLV-1 LTR luciferase reporter construct) as well as plasmids that express Tax, MEF-2A, HDAC9 and MEF-2A shRNA, was done as described in Methods. Before co-transfecting two or more plasmids, each of these plasmids was transfected alone to establish the background levels of luciferase activity. Cells were collected 24 hr post-transfection, lysed and assayed using the dual luciferase assay system. Firefly luciferase activity was normalized with that of *Renilla* luciferase expressed from phRL/CMV. Each bar represents the average of triplicate samples. Significance among groups was derived by student’s *t*-test to determine the p-value. (*p < 0.05). **(B)** MT-2 cells were transfected with either scrambled or shMEF-2 plasmid. Western blot analysis was performed at 24 hr and 48 hr to determine protein levels of MEF-2, Tax, and beta-actin. Data represent one of two separate experiments. **(C)** To analyze effects of shMEF-2A on virus production, transfected MT-2 cells were washed at 48 hr and incubated in fresh medium for another 24–36 hr. Thereafter, supernatants were assessed for HTLV-1 core protein levels (pg/ml) by the p19-specific ELISA (ZeptoMetrix, Buffalo, NY). **(D)** MT-2 cells were transfected either with a mock plasmid or MITR/HDAC9 plasmids followed by cell collection at every 24 hr over a 72 hr period. Real-time PCR analyses were performed to determine relative mRNA levels of Tax and p19. Data is representative of at least three independent experiments.

In order to understand effects of MEF-2 on transcriptional activation of HTLV-1 LTR, we transfected Jurkat cells with pU3R-luc (HTLV-1 5′ LTR luciferase reporter vector), and plasmids expressing Tax (pCMV-Tax), MEF-2A (p3X-Luc-MEF-2), and HDAC9 (pHDAC9). Also, the pLKO.1-puro shMEF-2A plasmid was used to knock down MEF-2A expression. As expected, LTR activity significantly increased in the presence of Tax (Figure 1A) Overexpression of MEF-2A, in the absence of Tax did not have any impact on LTR activation but showed slight enhancement on Tax activity with a p-value of 0.1. On the other hand, inhibition of MEF-2A expression by shRNA or its activity by HDAC9 demonstrated significant reduction in Tax-mediated LTR activation (Figure 1A), suggesting that Tax partners with cellular MEF-2 during the transactivation process. Each plasmid was titrated at various concentrations and was used at an optimal dose. Transfection efficiency was measured using a pMX-GFP plasmid (Lonza) and ranged from 58-64% in triplicate samples (Additional file 1: Figure S1A). Besides Tax plasmid no other plasmid has any direct effect on LTR activation providing internal control to the assay and avoiding the possibility of a general suppression with shMEF-2A and/or pHDAC9. For other controls, scrambled shRNA was used, MEF-2 inhibition was confirmed by end-point RT-PCR (Additional file 1: Figure S1B), and LDH cytotoxicity assay was performed to measure extracellular LDH in transfection media to confirm comparable viability of transfected cells among experimental variables (Additional file 1: Figure S1C).

Since the utilized dose of Tax plasmid exhibited limited LTR transactivation in Jurkat cells, we repeated some of the key observations in 293 T cells and observed an enhanced Tax activity, which was significantly reduced in the presence of both shMEF-2A and HDAC9 plasmids but not MEF-2A plasmid (Additional file 1: Figure S1D) in line with data obtained with Jurkat cells. As expected, MEF-2A knockdown alone in the absence of Tax had no effect on LTR activity avoiding the possibility of a general suppression.

Impact of MEF-2A shRNA and HDAC9 was further assessed in the setting of ongoing viral replication in MT-2 cells. First with an shRNA approach, we observed a reduction in Tax protein expression concurrent with decrease in MEF-2A expression upon shRNA administration into MT-2 cells (Figure 1B). This was also confirmed by FACS (data not shown) and translated at the level of virus production by MT-2 cells; wherein shMEF-2 demonstrated a significant (p < 0.001) reduction in p19 levels (Figure 1C). Next, upon HDAC9 overexpression (confirmed by Western blotting) in MT-2 cells, we noted a significant reduction in both Tax and p19 viral transcripts (Figure 1D). Tax downregulation was stable over a 72 hr period, with approximately 5-log down-regulation at 24 hr, 4.5-log at 48 hr, and 3-log downregulation at 72 hr. Importantly, we also observed p19 mRNA downregulation of approximately 4-log at 24 hr, 3.5-log at 48 hr, and 2.5-log at 72 hr with HDAC9 overexpression, confirming MEF-2 involvement in HTLV-1 LTR activation, subsequent Tax expression and viral replication as well as productive infection.

### MEF-2A is essential for HTLV-1-mediated T-cell proliferation

**Figure 2:**
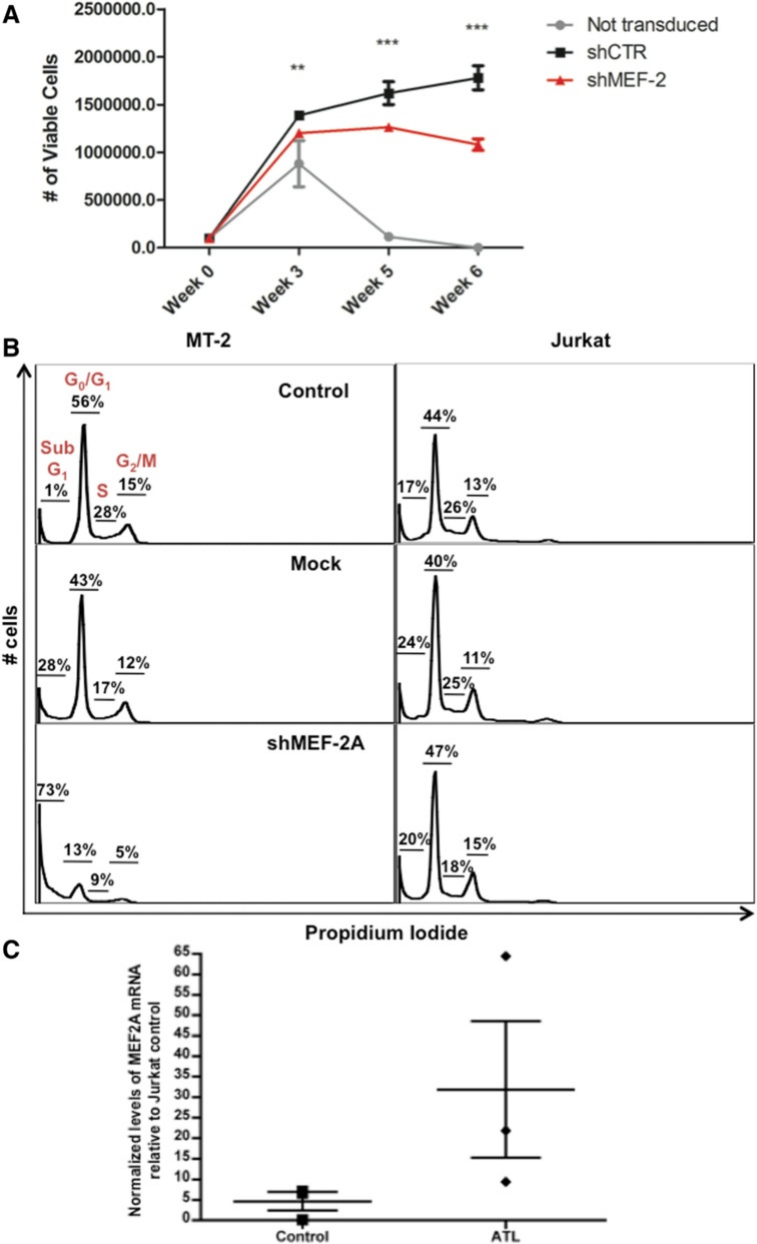
**MEF-2 inhibition perturbs HTLV-1-mediated T-cell transformation in correlation with a heightened MEF-2 expression in ATL patients. (A)** PBMCs were transduced with scrambled or shMEF-2 expressing lentivirus by spinoculation. Viable cell proliferation of PBMCs was determined by trypan blue exclusion assay, after co-culture with lethally irradiated MT-2 cells for the indicated times. Error bars represent standard deviation of triplicate samples with high significance (***p < 0.001) between shMEF-2 versus shControl samples. **(B)** MT-2 and Jurkat cells were collected at 24 hr post a 3-day transfection followed by staining with propidium iodide (PI-25 μg/ml, RNAase- 40 μg/ml, sodium citrate-0.1% and Triton-100 × −0.03%). Cell cycle progression of MT-2 and Jurkat cells were observed via flow cytometry with no transfection (upper panel), mock transfected (middle panel) and shMEF2 plasmid (2.5 μg/1×106 cells) transfection (lower panel). The percentage of sub-G1 cells, G0/G1, S and G2-M cells was analyzed using the FLowjo software. **(C)** MEF-2A mRNA levels was determined by the quantitative real-time PCR as described in Methods. At least two replicates per donor were processed and MEF-2 levels were compared between seronegative controls and ATL patients (n = 3, each). Each point represents average mRNA expression in individual donors. Bars represent mean with Standard Error (SEM) derived by a two-tailed, unpaired nonparametric *t*-*test* (Mann–Whitney).

In order to investigate whether MEF-2A is required for HTLV-1-induced transformation of T cells, we used a well-established co-culture model wherein HTLV-1 immortalizes primary T lymphocytes [64]. PBMCs were transduced with lentivirus expressing control scrambled or ShMEF-2A and subjected to a co-culture assay with MT-2 cells as the source of virus. Transduced cells were selected with puromycin after 3 weeks. As seen in Figure 2A, PBMCs transduced with shMEF-2A ceased proliferating at about 3 weeks whereas PBMCs expressing control shRNA continued cell growth and became immortalized. Control untransduced PBMCs were rapidly killed upon adding puromycin (Figure 2A). As expected, PBMCs by themselves without MT-2 did not survive long enough, thus it is difficult to determine from this assay whether MEF-2 is required for survival of cells in the absence of HTLV-1. Therefore, to further investigate this phenomenon, we determined changes in the cell cycle of Jurkat and MT-2 cells in the presence or absence of MEF-2 (shMEF-2A) by the PI-based assessment of DNA content in G0/G1, S, and G2/M phase. Early steps in cell death are characterized by internucleosomal DNA fragmentation and chromatin condensation, thus the apoptotic stage sub-G1 was also deduced. Figure 2B (left panel) displays a dramatic decrease of G0/G1 (13%) phase in shMEF-2A-transfected MT-2 cells compared to control- (56%) or mock transfected (43%). Concurrently, the cell population of the G2/M and M phases also decreased from 12% to 5% and those of S phase decreased from 12% to 9% in mock versus shMEF-2A conditions, respectively. This population reduction was concomitant with the emergence of a characteristic hypodiploid (<2 N DNA) sub-G1 peak, which indicates apoptotic cells. shMEF-2A containing cells showed a dramatic increase in the sub-G1 peak with 73% cells showing signs of undergoing apoptosis compared to 28% in the mock-transfected cells. Interestingly, this phenomenon of increased sub-G1 events was not observed in Jurkat cells (Figure 2B, right panel). For Jurkat, control, mock or shMEF-2A samples showed fairly similar percentages of cells in the G0/G1, S and G2/M phases. This data suggest that observed MEF-2 effects are specific to HTLV-1 and more so to Tax since lack of MEF-2A reduces LTR transactivation and subsequent Tax expression (Figure 1), consequently leading to cell cycle arrest and dispensing MT-2 cells of G0/G1 and G2/M phases to apoptosis.

The immortalization of primary T cells is a pathologic hallmark of ATL, and our data thus far suggested involvement of MEF-2A in this process. Interestingly, we observed a 7-fold increase in relative mRNA levels of MEF-2A in ATL patients as compared to seronegative control (Figure 2C) with a p*-*value of <0.18 in a two-tailed, unpaired nonparametric *t*-*test* (Mann–Whitney). Similar results were obtained while comparing MEF-2A levels in ATL patients with silent carriers of virus establishing clinical relevance of this cellular factor in HTLV-1-associated disease pathologies. A heightened MEF-2A expression in ATL patients could suggest a direct role of MEF-2A in the genesis and/or maintenance of T-cell leukemia in these patients.

### MEF-2A is recruited to the HTLV-1 LTR in the context of chromatin

**Figure 3:**
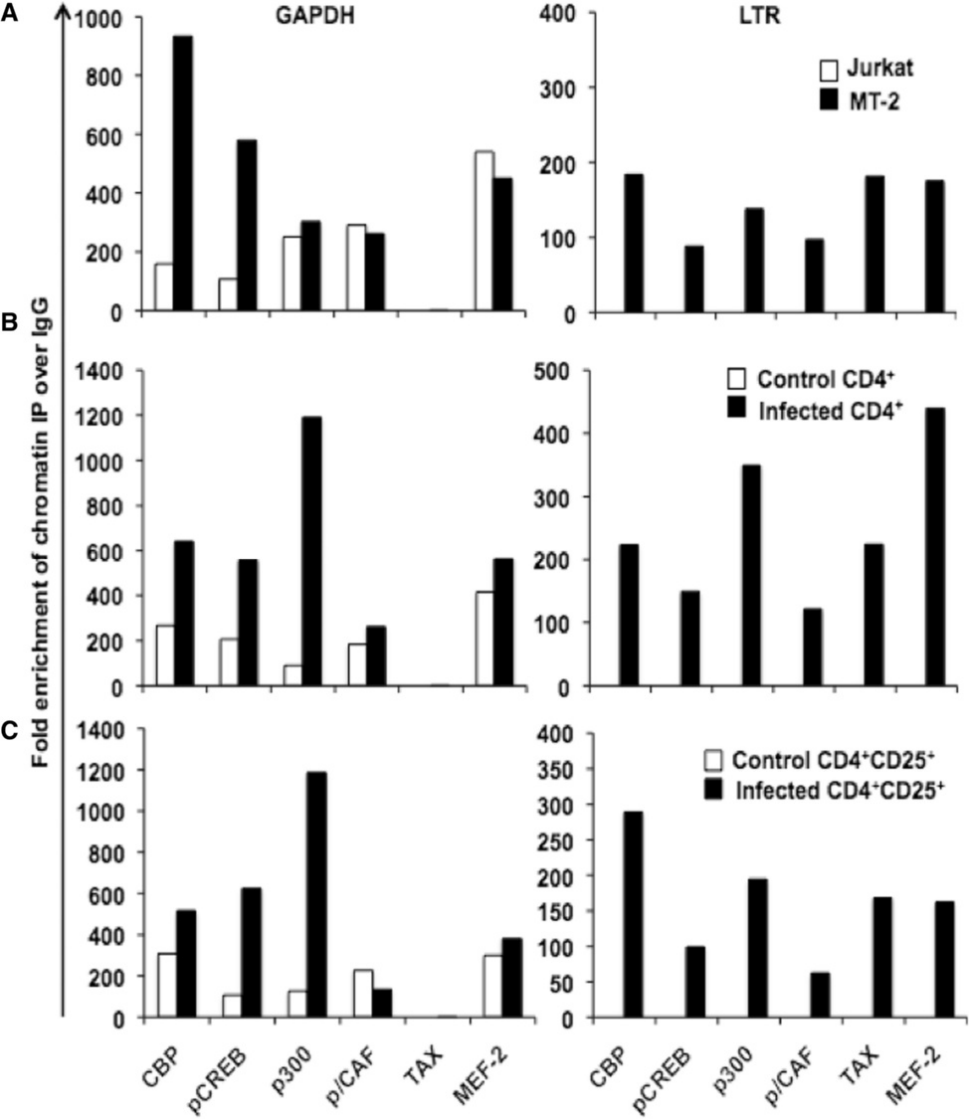
**Tax and MEF-2 are recruited to the HTLV-1 LTR.** Chromatin immunoprecipitation of Tax protein and transcription factors bound to cellular and viral promoters during HTLV-1 infection in **(A)** cell lines, **(B)** primary CD4^+^ T cells, and **(C)** primary CD4^+^CD25^+^ T cells was performed using the ChIP-IT Express kit. Cells were lysed in a dounce homogenizer to obtain sheared chromatin following formaldehyde fixation. The sheared chromatin was immunoprecipitated at 4°C overnight using 2 μg of antibodies against the Tax protein, indicated cellular factors and controls. The immunoprecipitated chromatin was then subjected to PCR using primers for HTLV-1 LTR and human GAPDH. Data is presented as average fold change over control IgG immunoprecipitation, and is representative of three independent experiments.

Having generated confidence in MEF-2 involvement in HTLV-1 pathogenesis, we proceeded to understand the underlying molecular interactions in the context of primary CD4^+^ T cells and viral infection. We infected primary CD4^+^ T cells with HTLV-1 as previously described [65,66], and confirmed intracellular Tax expression by flow cytometry as well as by Western blotting (Additional file 2: Figure S2). Upon confirmation of infection, cells were subjected to ChIP analyses. In both cell lines and primary cells, we noted strong binding of CBP, pCREB, p300, p/CAF, and MEF-2A but not Tax to the GAPDH promoter (Figure 3, left panels). This was not surprising since the amplified region of GAPDH contained binding sites for these TFs. Although recruitment of some of these factors to the GAPDH promoter was more efficient in infected cells, we did not see any increase in GAPDH expression upon HTLV-1 infection (Additional file 3: Figure S3). We also observed efficient recruitment of TFs and Tax to the viral LTR in MT-2 cells (Figure 3A, right panel) and infected CD4^+^ cells (Figure 3B, right panel), but not in uninfected control cells. CD4^+^CD25^+^ T cells were also included in our analysis, as they are the primary subset of CD4^+^ T cells targeted by HTLV-1 [67]. These cells showed efficient recruitment of MEF-2A and other cellular factors to the LTR upon infection (Figure 3C, right panel). As a control, we enriched cells for viral core protein p19 and as expected did not detect recruitment of any factors analyzed to GAPDH or LTR promoters (Additional file 4: Figure S4). Altogether, these results confirmed that MEF-2A is recruited to the HTLV-1 LTR in association with Tax and co-activators of transcription including p300, CBP, and p/CAF.

### MEF-2 is upregulated upon HTLV-1 infection and physically interacts with Tax

**Figure 4:**
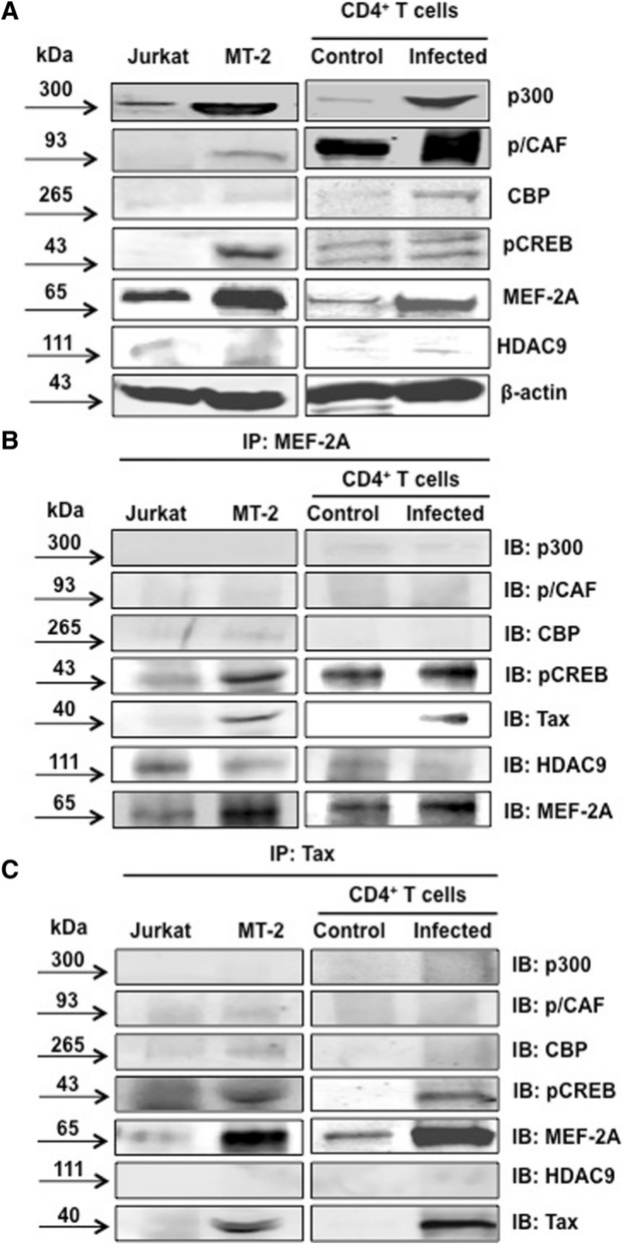
**MEF-2 physically interacts with Tax. (A)** Control (Jurkat), infected (MT-2) cell lines, control primary CD4^+^ T cells and HTLV-infected primary CD4^+^ T cells were lysed, sonicated and protein concentration was determined by Bradford assay. Equal protein quantities were then resolved by SDS-PAGE and transferred to PVDF membrane. Following a 1 hr block membranes were incubated with antibodies against the transcription factors. Western blot shows the expression of transcription factors in control and infected cell lines and primary cells. **(B)** MEF-2 complex formation with Tax and transcription factors was analyzed using an immunoprecipitation assay. Cells were lysed using an immunoprecipitation lysis buffer and then incubated with MEF-2 antibody overnight at 4°C as described in Methods. Western immunoblot analysis was performed to confirm immunoprecipitation. **(C)** Control and infected cell lines and primary cells were enriched for Tax and immunoblotted to determine complex formation with MEF-2 and transcription factors. Data is representative of multiple individual experiments.

Prior to protein-protein interaction studies, we examined the expression of MEF-2A and other cellular factors both in cell lines and primary cells without and with HTLV-1 infection. As shown in Figure 4A, we noticed an upregulation of the HATs p300, CBP and p/CAF, as well as TFs, pCREB and MEF-2A upon infection. We also observed the complex formation of MEF-2A with Tax and pCREB, confirming a direct interaction with the Tax/CREB heterodimer complex (Figure 4B). Interestingly, upon infection, the interaction of MEF-2A with HDAC9 was expectedly diminished since HDAC9 binds to the C-terminal TAD domain of MEF-2 to repress its transcriptional activity. MEF-2A direct interaction with Tax was confirmed while enriching for Tax, which coprecipitated both pCREB and MEF-2A (Figure 4C). Tax enriched samples contain some levels of HATs but not HDAC9 suggesting that Tax can directly interact with co-activators of transcription as shown before [68]. It appeared that anti-Tax nonspecifically pulled down some MEF-2 in the absence of Tax; however, the band was greatly increased in the presence of Tax. Nevertheless, we performed an alternative experiment to validate the interaction of these two proteins in C8166 cells, which contain both Tax and MEF-2 proteins in abundance and demonstrated a specific interaction of these two proteins (Additional file 5: Figure S5A).

In addition, we transfected 293 T cells (upon detecting MEF-2 presence in these cells) with the FLAG-Tax expression plasmid and confirmed the expression of Tax in the lysate. We then precipitated epitope-tagged Tax with the anti-FLAG antibody and performed Western blotting with the anti-MEF-2 antibody, which led to the detection of a specific band in FLAG-Tax transfected but not mock transfected sample (Additional file 5: Figure S5B).

### MEF-2 co-localizes with Tax

**Figure 5:**
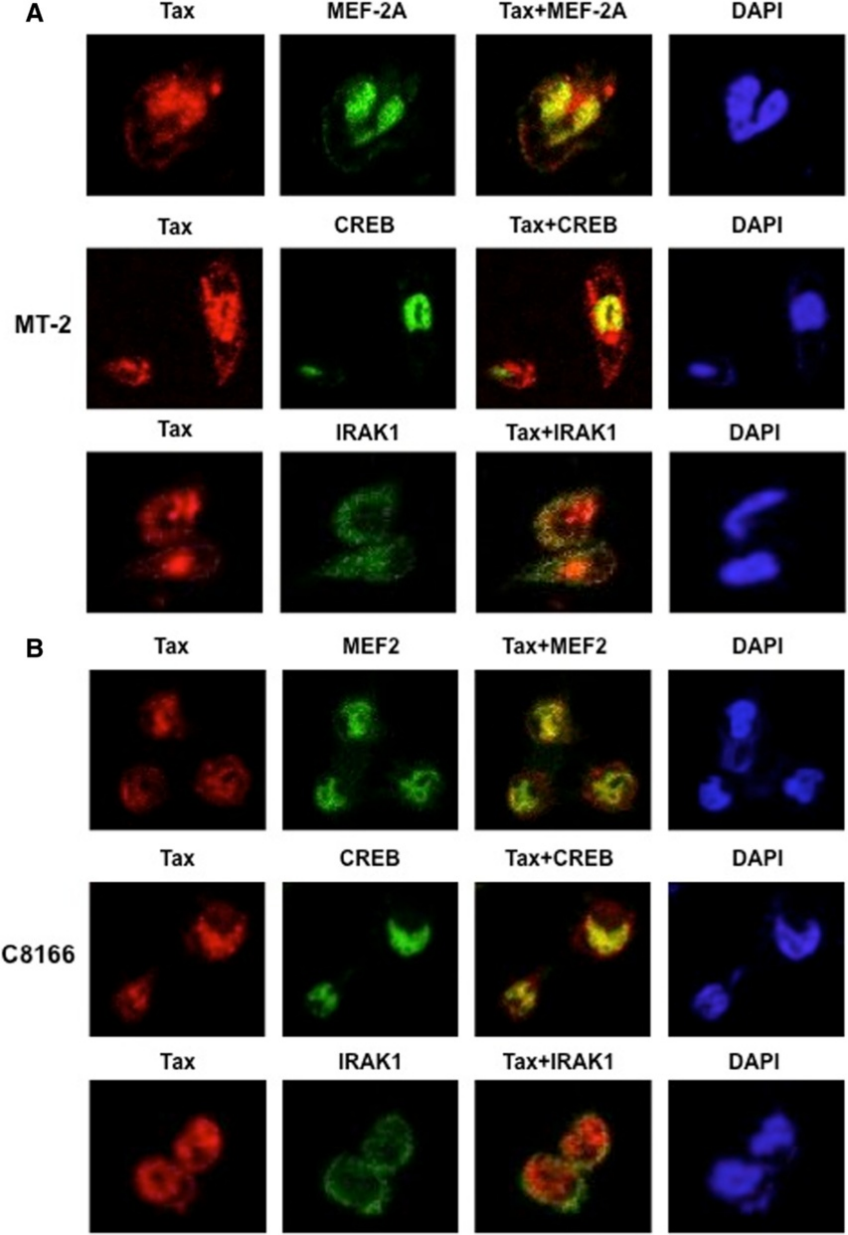
**MEF-2 co-localizes with Tax in the nucleus of HTLV-1 transformed cell lines.** MT-2 cells **(A)** and C8166 cells **(B)** were cultured overnight on poly-L-lysine coated glass coverslips. The cells were then fixed, permeabilized and then stained with anti-Tax, anti-MEF-2, anti-CREB or anti-IRAK1 as indicated. Nuclei were stained with DAPI and cells were subjected to confocal microscopy.

To confirm that MEF-2 interacts with Tax not only in solution but also within the infected cells, we performed confocal microscopy using two HTLV-1 transformed cell lines, MT-2 (Figure 5A) and C8166 (Figure 5B). Tax localized in the cytoplasm and nucleus in both cell lines. pCREB as a positive control strongly co-localized with Tax in the nucleus. Tax and MEF-2A also co-localized within the nuclear compartment. As a negative control, a cytoplasmic protein (IRAK1) without any known interaction with Tax was used. As expected, this protein did not co-localize with Tax in either cell type.

Altogether, these results clearly showed a direct interaction of MEF-2A with the Tax/CREB heterodimer complex suggesting that MEF-2 may facilitate the stabilization of the complex between Tax and CREB on the HTLV-1 LTR in addition to directly participating in the transactivation process. This was confirmed by knocking down MEF-2A in MT-2 cells and testing for the Tax/CREB recruitment to the LTR by a novel promoter-binding assay.

### HTLV-1 LTR transcriptional activation complexes contain MEF-2

**Figure 6:**
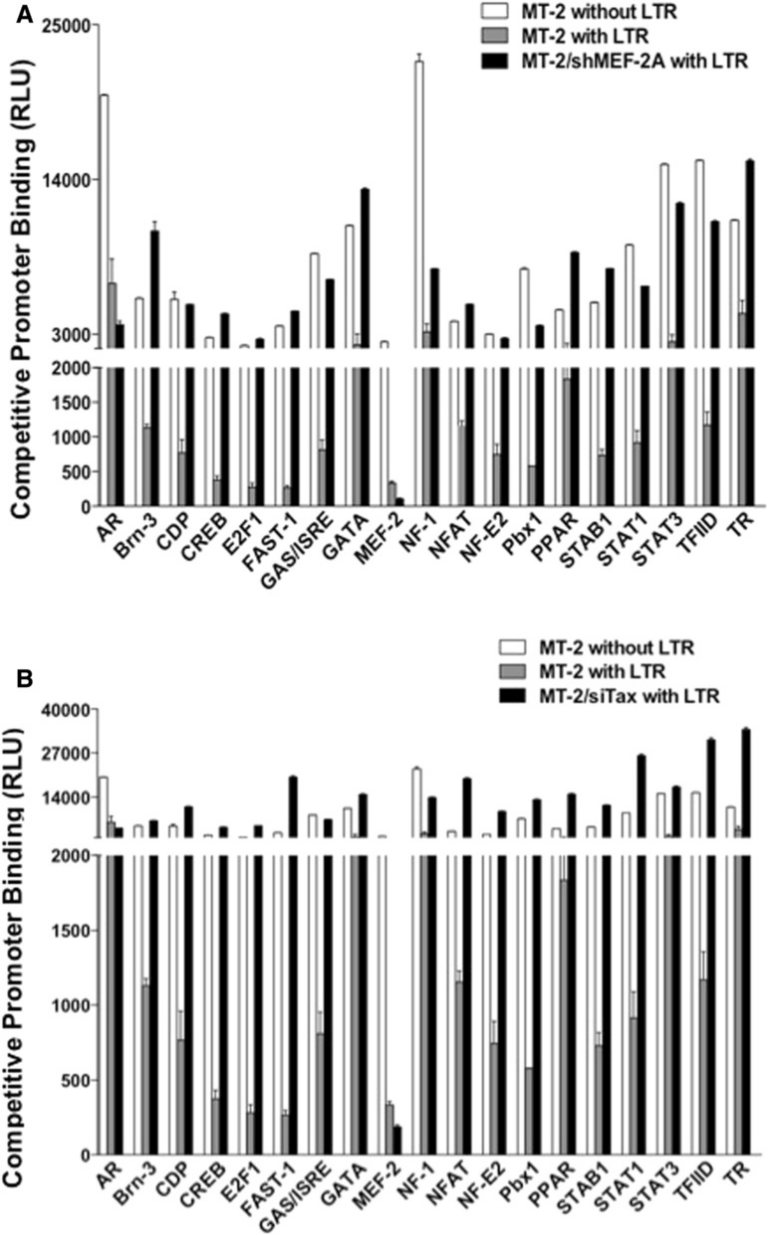
**HTLV-1 LTR transcriptional activation complexes contain MEF-2.** Binding of various transcription factors present in the nuclear extract isolated from MT-2 cells to the HTLV-1 LTR was assessed using the Promoter-Binding Transcription Factor Profiling Array I upon knocking down MEF-2A **(A)** and Tax **(B)** as described in Methods. The nuclear extract was incubated with oligonucleotide probe mix with the HTLV-1 LTR or a control DNA. The binding of each transcription factor to LTR was indicated by average reduction in chemiluminescence of transcription factor-specific oligonucleotide probe specific to each factor from triplicate samples.

In order to confirm that HTLV-1 LTR transcriptional complexes contain MEF-2, we performed a promoter-binding TF profiling assay. Use of this assay offers the advantage of analyzing multiple TFs at once as opposed to electrophoretic mobility shift assay, which enables characterization of only a single TF at a time. This assay is based upon the fact that if a TF binds the LTR, then the binding of that factor with its oligonucleotide is reduced due to competition between the probe and LTR. Of the 50 total TFs, only 19 including MEF-2 showed an ability to compete for binding to the LTR at significant levels (Figure 6). Observed binding of CREB validated the assay, since it is an established factor that interacts with Tax and facilitates transactivation process [29,69-71]. Some other cellular factors were identified (i.e. AR, Brn-3, Pbx1, etc.) whose roles remain to be tested in HTLV-1 pathogenesis. Similarly, 31 cellular factors (i.e. ATF2, CAR, EGR, etc.) did not show significant competitive inhibition of LTR binding within MT-2 cells. These observations were made in the setting of active infection where both Tax and MEF-2 were readily available to mediate the process.

Upon inhibition of both MEF-2A (Figure 6A) and Tax (Figure 6B), almost all TFs (except AR) lost their binding to LTR and some were present in much higher levels compared to control (MT-2 cells without any inhibition and exogenous LTR). Inhibition of Tax or MEF-2 affected each other protein levels (confirmed by Western and FACS). In both cases, MEF-2 was not present in the nuclear extract; therefore, a majority of MEF-2 probe molecules in the assay remained unbound and were washed off leading to a low signal. While other cellular factors being freed from the LTR exhibited higher signals depending upon their relative abundance in the nuclear extract. Of these factors, nuclear factor of activated T cells (NFAT) was found to be of great significance. NFAT, upon activation, translocates into the nucleus and cooperates with various transcription factors including MEF-2 to promote their transcriptional activity [62,72]. In accordance, LTR-binding of NFAT was abrogated not only in the absence of Tax (Figure 6B) but also MEF-2 (Figure 6A). The dephosphorylation and activation of NFAT is mediated by a calcium-dependent serine-threonine phosphatase, called Calcineurin. Tax has been shown to activate calcineurin [73-75], and its inhibition in MT-2 cells resulted in significant reduction in the calcineurin activity (Additional file 6: Figure S6). Thus physical interaction between NFAT and MEF-2 protein provides a mechanism for Tax-mediated activation of MEF-2 via calcineurin signaling. Additional file 7: Table S1 provides intensity values (raw and average) of all analyzed TFs in each condition.

### MEF-2 directly binds to the HTLV-1 LTR and regulates Tax activation of the LTR

**Figure 7:**
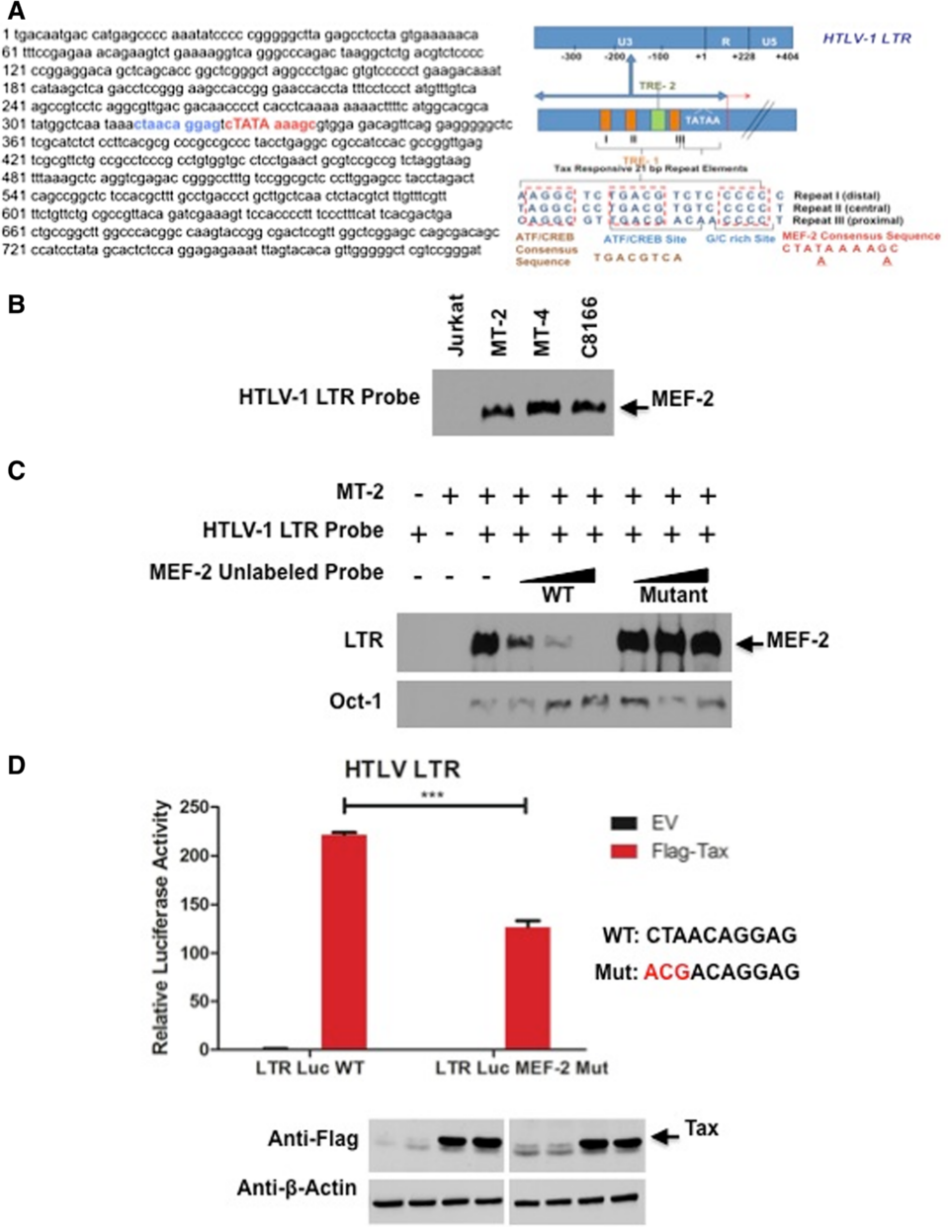
**Assessment of MEF-2 binding site(s) within the HTLV-1 LTR. (A)** HTLV-1 LTR nucleotide sequence with two putative MEF-2 binding sites shown in blue and red. The HTLV-1 LTR comprises U3 (unique 3′), R (Repeated), and U5 (unique 5′) regions. The U3 region regulates viral gene expression via three 21 bp repeats known as Tax-responsive element - 1 (TRE - 1), which confers Tax-based *trans*-activation. These repeats contain 3 conserved domains labeled A, B and C. The location for ATF/CREB binding and putative MEF-2 binding are illustrated. **(B)** EMSA was performed with a probe corresponding to the MEF-2 site in the HTLV-1 LTR using nuclear extracts from Jurkat, MT-2, MT-4 and C8166 cells. **(C)** EMSA competition assay was performed using nuclear extracts from MT-2 cells, with increasing amounts of unlabeled consensus MEF-2 specific probe (200, 300 and 400 fold molar excess respectively) or a mutated MEF-2 specific probe. Oct-1 was used as a loading control. **(D)** HTLV-1 LTR luciferase assays in 293 T cells transfected with empty vector (EV) or Flag-Tax using LTR luciferase WT plasmid (LTR Luc WT) or a MEF-2-specific binding mutant (LTR Luc MEF-2 Mut). Luciferase values are presented as “fold induction” relative to the control (EV). Two-tailed unpaired *t-test* was performed with Prism software. Error bars represent the standard deviation of triplicate samples. The level of significance was defined as: ***p < 0.001. Western blots were performed with anti-Flag and anti-β-actin using whole-cell lysates.

Results from the competitive promoter assays and CHIP assays confirmed that MEF-2 is recruited to the HTLV-1 LTR suggesting the presence of a MEF-2 binding site(s) within the LTR. Therefore, we examined the consensus site for all MEF-2 isoforms CTA(T/A)4TA(G/A)C within the LTR and identified two imperfect MEF-2 sites - one just downstream of repeat III of the Tax-response element (TRE) and upstream of the TATA box (Figure 7A, blue highlight) that matches 7/10 bases of the consensus site; the other overlaps with the TATA box (Figure 7B, red highlight), which is highly unlikely to be functional due to competition with general TFs. To determine if MEF-2 interacted with the more upstream MEF-2 site in the HTLV-1 LTR, a double-stranded DNA probe corresponding to this site was generated and used for EMSA DNA binding assays. EMSA revealed a specific protein/DNA complex with nuclear extracts from the HTLV-1 transformed cell lines MT-2, MT-4 and C8166, but not Jurkat cells (Figure 7B). To demonstrate that this protein complex consisted of MEF-2, competition EMSA assays were performed with 200–400 fold molar excess of unlabeled consensus MEF-2 probe or a mutated MEF-2 probe. As seen in Figure 7C, unlabelled consensus MEF-2, but not the mutant form, effectively competed with the MEF-2 probe derived from the HTLV-1 LTR for protein binding. This result strongly supports the idea that MEF-2 directly binds to the imperfect MEF-2 site in the HTLV-1 LTR, just upstream of the TATA box. To ascertain the functional significance of this MEF-2 site, the first three nucleotides were mutated (CTA- > ACG) in the context of the HTLV-1 LTR luciferase reporter. Luciferase assays were conducted to examine Tax activation of a wild-type HTLV-1 LTR and the MEF-2 mutated LTR. As expected, Tax strongly activated the HTLV-1 LTR; however, Tax-induced activation of the MEF-2 mutant LTR was significantly decreased even though Tax expression was similar (Figure 7D). Together, these results indicate that MEF-2 directly binds to DNA in the HTLV-1 LTR and is important for Tax activation of the LTR.

### MEF-2 associated signaling pathways are triggered by HTLV-1 infection

**Figure 8:**
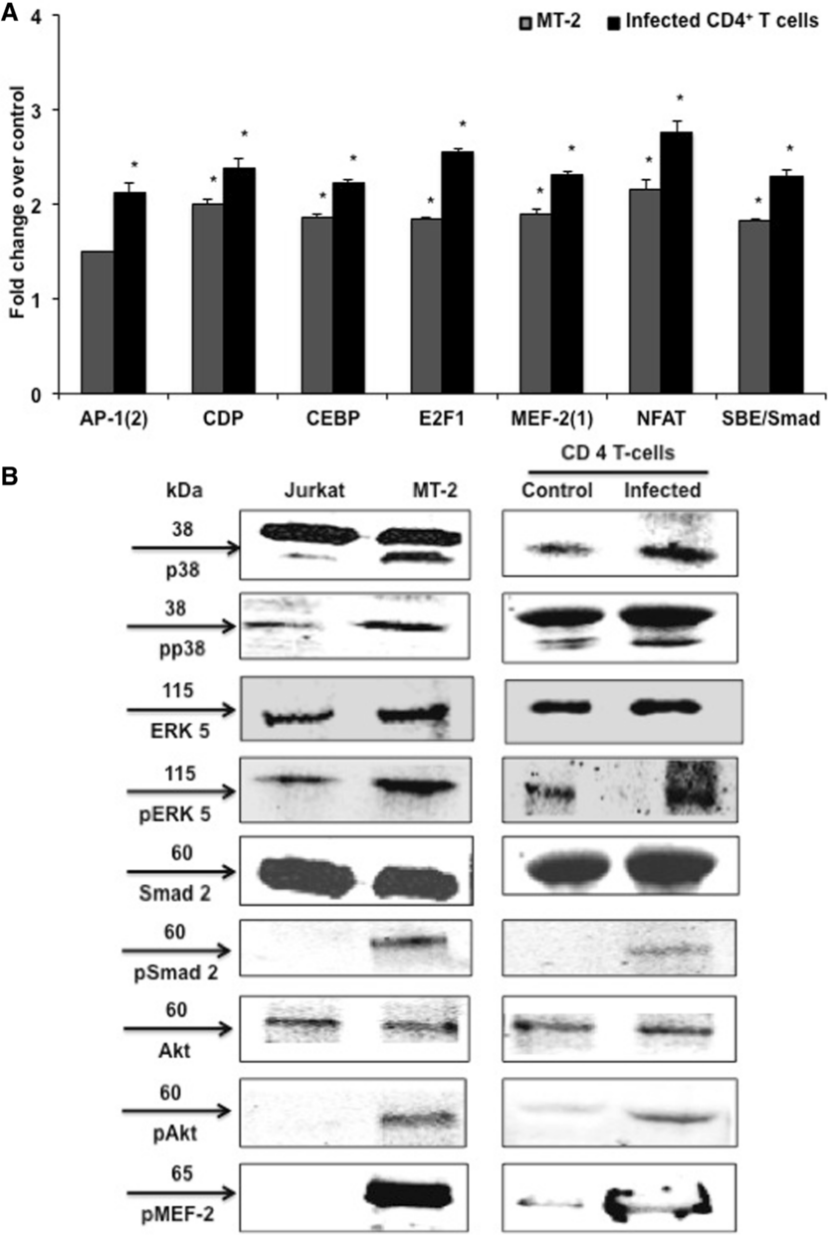
**MEF-2-associated signaling pathways are triggered during HTLV-1 infection. (A)** A protein-DNA array was used to determine the activation of the various eukaryotic transcription factors. Nuclear extracts from control and HTLV-infected primary cells were mixed with biotinylated DNA binding oligonucleotides for the formation of protein-DNA complexes. These probes were then hybridized to pretreated array membranes and the bound probe was detected as described in Methods. Densitometric analysis was used to quantify the spots and data was normalized to their respective controls after background subtraction. Fold change in expression of selected transcription factors from the array data compared to relevant uninfected cells. Significance was determined using the Student’s *t-test* (*P ≤ 0.05). **(B)** Cells were lysed and Western blotting was performed to confirm the array data.

**Figure 9:**
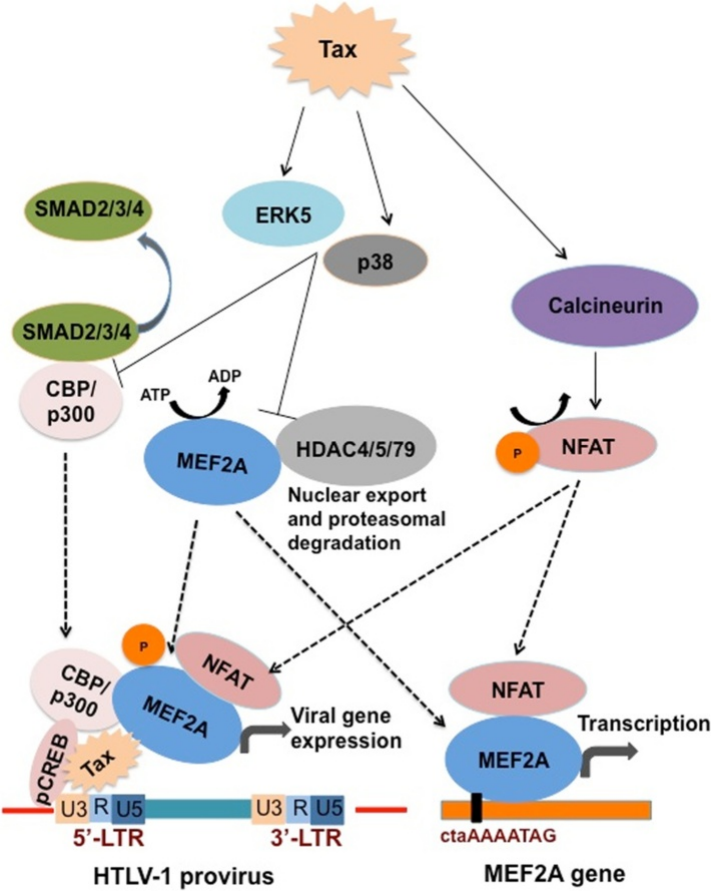
**Model explaining MEF-2 activity on Tax-mediated transactivation of HTLV-1 LTR.** Type II HDACs (HDAC4/5/7/9) bind to MEF-2A and repress its transcriptional activity. Upon HTLV-1 infection, Tax activates p38 and ERK5, which phosphorylate MEF-2 leading to its dissociation from the MEF-2A: HDAC repressive complex. On the other hand, Tax also binds to Smad2/3/4 to prevent their constitutive binding to transcription co-activators CBP/p300. This leads to increased availability of CBP/p300 to bind Tax/pCREB complex bound to the 5′ LTR region of the provirus. Along with Tax/pCREB/CBP/p300 complex, recruitment of MEF-2A to the 5′ LTR promotes viral gene expression. Tax also activates Calcineurin (a calcium-dependent serine-threonine phosphatase), which dephosphorylates NFAT. Upon dephosphorylation, NFAT translocates to nucleus and is recruited to the HTLV-1 5′ LTR along with the Tax/pCREB/CBP/p300 complex. NFAT is also recruited to the MEF-2A gene promoter where it binds to MEF-2A and turns on transcription resulting in upregulation of MEF-2A expression. HDAC, Histone deacetylase; MEF-2A, Myocyte-specific enhancer factor 2A; ERK5, Extracellular-signal-regulated kinase 5; Smad, Sma- and Mad-Related Protein; CREB, cAMP response element-binding protein; CBP, CREB-binding protein; NFAT, Nuclear factor of activated T cells.

The transcriptional activity of MEF-2 is regulated by protein modifications including phosphorylation, acetylation, sumoylation, etc. In addition, MEF-2 is known to integrate a number of signaling pathways, including PI3K/Akt, NF-κB, MAPK, TGF-β and JAK/STAT signaling [33,38,76]. While many of these signaling pathways are known to be activated by Tax, we obtained a global perspective on these signaling events upon HTLV-1 infection of CD4 T cells by a DNA-protein array. This type of interactome profiling of primary CD4 T cells upon HTLV-1 infection has not been reported yet. In general, an upregulated transcriptome profile was seen in both MT-2 and primary infected CD4^+^ T cells, emphasizing the highly active and dynamic process of viral infection (Additional file 8: Figure S7A and B). For analysis, cellular factors included in the array were grouped according to their association with relevant signaling pathways in Table 1 and the fold-change in protein expression of the key cellular factors from the MEF-2-integrated signaling cascades is given in Figure 8A. Upon validation of array data by Western blotting, the phosphorylated form of MEF-2A as well as of p38, ERK5, Smad2 and Akt were found to be upregulated upon infection in both MT-2 and primary cells (Figure 8B). Thus, our model (Figure 9) suggests that Tax-mediated activation of cellular signaling pathways contribute to the phosphorylation and activation of MEF-2, which is then dissociated from class II HDACs and interacts with Tax at the viral promoter to boost Tax-mediated transactivation, viral replication as well as T-cell transformation. Tax also binds to Smad2/3/4 to prevent their constitutive binding to transcription co-activators CBP/p300. This leads to increased availability of CBP/p300 to bind Tax/pCREB complex bound to the 5′ LTR region of the provirus. Along with Tax/pCREB/CBP/p300 complex, recruitment of MEF-2A to the 5′ LTR promotes viral gene expression. On the other hand, Tax also activates Calcineurin (a calcium-dependent serine-threonine phosphatase), which dephosphorylates NFAT. Upon dephosphorylation, NFAT translocates to the nucleus and is recruited to the HTLV-1 LTR along with the Tax/pCREB/CBP/p300 complex. NFAT is also recruited to the MEF-2A gene promoter where it binds to MEF-2A and turns on transcription resulting in upregulation of MEF-2A expression. Interestingly, in Figure 4A we showed that MEF-2 in its native form was also upregulated upon HTLV-1 infection making more MEF-2 available for interaction with Tax. This heightened expression of MEF-2 could be a result of Tax-induced calcineurin activity that in turn results in NFAT-mediated MEF-2 transcription via binding to its own promoter.

**Table 1:** **Raw densities of the transcription factors included in the Protein/DNA array analysis, grouped according to association with general signaling pathways**

		**Raw densities**			
**Signaling pathways**	**Transcription factors**	**Jurkat**	**MT-2**	**Control CD4**	**Infected CD4 T cells**
Akt signaling	AP-(2)	29011 ± 312	36283 ± 2049	126 ± 3	285 ± 2
	Brn-3	20098 ± 857	43533 ± 350	0	262 ± 2
	GATA	12325 ± 357	34756 ± 1662	0	284 ± 1
	NFAT	16264 ± 921	35151 ± 358	103 ± 2	284 ± 1
	NF-E1	27661 ± 910	45077 ± 611	120 ± 1	326 ± 3
	PAX-5	26980 ± 1038	33945 ± 97	120 ± 1	261 ± 2
	Pit-1	11622 ± 386	29345 ± 283	0	296 ± 3
	PPAR	13329 ± 467	31100 ± 82	107 ± 2	264 ± 1
	PRE	14780 ± 196	30151 ± 417	109 ± 0	263 ± 3
	TR	23845 ± 973	3172 ± 231	0	296 ± 3
MAP kinase signaling	AP-1(2)	26548 ± 1383	39830 ± 2086	133 ± 8	284 ± 1
	AP-1(1)	19105 ± 524	45066 ± 1252	100 ± 1	284 ± 1.6
	CDP	14854 ± 434	29735 ± 75	106 ± 2	253 ± 2
	CEBP	20286 ± 68	37664 ± 693	115 ± 3	257 ± 2
	E2F1	25614 ± 251	47332 ± 149	126 ± 6	323 ± 8
	ER	30792 ± 1518	42869 ± 335	127 ± 7	284 ± 4
	Ets	17337 ± 489	32485 ± 1058	114 ± 1	265 ± 3
	IRF-1	13996 ± 124	26838 ± 30	102 ± 1	241 ± 2
	MEF-1(1)	23260 ± 405	47037 ± 954	133 ± 1	287 ± 2
	MEF-2(1)	15230 ± 107	28955 ± 492	105 ± 2	244 ± 5
	c-Myb	23884 ± 1150	32938 ± 656	118 ± 0	270 ± 2
	NF-1	14970 ± 920	28197 ± 45	114 ± 1	258 ± 5
	Pbx-1	13464 ± 336	29920 ± 67	109 ± 0	245 ± 4
	p53	24993 ± 372	31927 ± 89	120 ± 0	270 ± 2
	USF-1	277010 ± 272	34861 ± 171	117 ± 2	285 ± 0
	VDR/DR-3	26164 ± 145	32870 ± 246	117 ± 0	281 ± 1
NF-kB signaling	HSE	15326 ± 371	30604 ± 440	111 ± 0	263 ± 5
	NF-E2	28464 ± 800	39988 ± 2116	117 ± 2	307 ± 4
JAK/STAT signaling	GAS/ISRE	12857 ± 427	34972 ± 566	109 ± 4	238 ± 10
	HNF-4	19187 ± 108	42759 ± 1490	116 ± 1	281 ± 1
	STAT 1	15351 ± 156	32659 ± 157	116 ± 1	294 ± 5
	STAT 3	17637 ± 567	33608 ± 142	116 ± 2	0
	STAT 4	29321 ± 608	37269 ± 462	126 ± 2	270 ± 0
TGF-β signaling	FAST-1	15054 ± 756	33012 ± 477	116 ± 0	294 ± 5
	Myc/Max	26136 ± 1301	0	0	0
	RAR/DR-5	28178 ± 45	36611 ± 97	117 ± 0	275 ± 2
	SBE/Smad	16635 ± 511	30441 ± 626	108 ± 1	248 ± 5

## Discussion

While the precise molecular mechanisms that influence HTLV-1 clinical latency versus active disease remain poorly understood, it is well established that Tax is essential for efficient HTLV gene expression [11-13,70,77]. Tax-mediated transcriptional regulation occurs via direct interaction with the CREB homodimer to form a stable Tax/CREB complex [21-24,27-29]. Complex binding at TREs within the viral promoter initiates HAT recruitment to facilitate nucleosome acetylation and disassembly, thus enabling active viral transcription [19,25,26,29]. Previously we showed a differential requirement for chromatin remodeling machinery in human T cells with stably versus transiently integrated viral LTR [69]. Herein, we provide evidence of recruitment of a novel cellular factor, MEF-2, to the LTR and its role in viral pathogenesis.

Although, it is considered a transcriptional activator, the regulatory activity of MEF-2 is determined by its secondary interactions. MEF-2 remains constitutively bound to target gene promoter regions, but is held transcriptionally silent through associations with class II HDAC complexes that maintain the chromatin in a condensed, hypoacetylated state [35,38,76]. In the unstimulated state, HDAC9 interacts with MEF-2 and represses its activity; whereas upon activation of intracellular signaling pathways involving Ca2+/calmodulin-dependent kinase (CaMK) and protein kinase D, it undergoes CRM1-dependent nuclear export [38,78,79]. Besides this, other regulatory mechanisms, such as sumoylation [80,81], caspase cleavage [82,83], ubiquitin-dependent proteosomal degradation [84,85], and mitochondrial targeting [86], have also been reported for negative regulation of class IIa HDAC activity. In contrast, MEF-2 can also get activated through direct modification by p38 MAP kinase and ERK5-mediated phosphorylation [44,49,55-60]. Phosphorylation of MEF-2A at Thr312 and Thr319 within the transcription activation domain by p38 enhances MEF-2A: MEF-2D heterodimer-dependent gene expression [49]. HTLV-1 Tax has been shown to activate p38 and other MAP kinases [53,54]. Tax also operates at another level to promote Tax-mediated transcriptional activation of the viral LTR. Tax binds the N-terminus of Smad2, Smad3, and Smad4 proteins, which inhibits their association with Smad-binding elements and co-activators CBP/P300 [87]. This causes an increase in availability of CBP/P300 to bind to Tax leading to increased viral gene expression. Herein, we demonstrated successful inhibition of MEF-2 and its consequence on Tax-mediated LTR activation (Figure 1A), viral gene expression (Figure 1B), viral replication (Figure 1C), and CD4 T-cell transformation (Figure 2A-B). Physiological relevance of MEF-2 was also established as ATL patients expressed more MEF-2 compared to controls (Figure 2C). MEF-2 expression was inhibited by an shRNA specific for MEF-2A and its activity was inhibited by the MITR plasmid that encodes HDAC9. The ubiquitous nature of MEF-2 makes it difficult to achieve an efficient knockdown in expression; however, HDAC9 efficiently represses the transcriptional activation of MEF-2 [35,88-90]. While shRNA was specific for MEF-2 isoform “A”, HDAC9 is known to repress transcriptional activity of all MEF-2 isoforms [35,88-90]; however, similar levels of inhibitions was achieved by both in Figure 1A suggesting that MEF-2A could be the major player in Tax activity on HTLV-1 LTR. Upon confirmation of MEF-2 importance in HTLV-1 pathogenesis we investigated the underlying molecular processes as well as MEF-2 regulation by Tax and cellular factors (Figures 3, 4, 5, 6, 7 and 8). The model given in Figure 9 details our view about how MEF-2 activity is regulated during HTLV-1 infection. Tax activates a multitude of cellular signaling pathways, including those integrated by MEF-2. To our surprise, previously published interactome studies for HTLV-1 [91] or Tax interactome [68,92], did not identify MEF-2 as a potential player thereby enabling presented studies to be the first in this line.

MEF-2 has shown to be required for B cell proliferation and survival after antigen receptor stimulation [93] and MEF-2A is suggested to play a role in thymocyte differentiation [94]. MEF-2C deficiency is associated with defects in the production of B cells, T cells, natural killer cells and common lymphoid progenitor cells [95]. Tax-mediated recruitment of MEF-2 to the viral promoter may capitalize on these conserved mechanisms to activate viral transcription. For example, in thymocyte negative selection, expression of Nur77 is dependent on MEF-2; while MEF-2 remains constitutively bound to the promoter, it is held transcriptionally silent by interactions with Cabin1, which recruits the co-repressor mSin3a in association with class II HDACs [38,40,41,96-100]. However, increases in intracellular calcium levels activate the mobilization of calcineurin and calmodulin, which displace the Cabin1/mSin3a/HDAC inhibitory complex and allow p300, p/CAF and CBP binding. MEF-2 is known to integrate a number of overlapping cellular signaling pathways that are also influenced by calcium signaling. This study and previous ones have shown that Tax activates Calcineurin, which dephosphorylates NFAT. Upon dephosphorylation, NFAT translocates to the nucleus and is recruited to the HTLV-1 5′ LTR along with the Tax/pCREB/CBP/p300 complex. NFAT is also recruited to the MEF-2A gene promoter where it turns on transcription resulting in upregulation of MEF-2 expression.

## Conclusions

Our study provides the first evidence for the involvement of MEF-2 in Tax-mediated LTR activation, viral replication, and T-cell transformation. The relevance of this finding is established by the fact that we found an increased expression of MEF-2 in ATL patients compared to controls. The underlying molecular mechanisms include the direct binding of MEF-2 to DNA within the HTLV-1 LTR in the context of chromatin and the MEF-2 dependent co-regulation of transcriptional complex involving both Tax and CREB. MEF-2 activity may be regulated by MEF-2 integrated signaling pathways that were activated during HTLV-1 infection of primary CD4^+^ T cells.

## Methods

### Cell lines and plasmids

The HTLV-1-transformed cell line C8166 [101], and HTLV-1 producing cell lines, MT-2 [102] and MT-4 are described previously [103,104]. Jurkat cells, a human CD4 T cell line and Human embryonic kidney cells (HEK 293 T) were purchased from ATCC. Expression vectors encoding pU3R-Luc (LTR-Luc), pRL-TK (thymidine kinase), pCMV-Tax and Tax M47 have all been described previously [105,106]. MEF-2 expression plasmid 32958 (pCGN-MEF2A-HA), and a luciferase reporter plasmid 32967 (p3X-Luc-MEF-2A) were purchased through Addgene (Cambridge, MA). The control scrambled and MEF-2A shRNA (shMEF-2) plasmids were purchased through Sigma-Aldrich (St. Louis, MO). The plasmid DNA encoding the MEF-2 interacting transcriptional repressor (MITR/HDAC9) was purchased through the Dana-Farber/Harvard Cancer Center DNA Resource Core (PlasmID). LTR Luc MEF-2 mutant was generated using the QuikChange® Site-directed mutagenesis kit (Agilent Technologies) with the following primers: forward/reverse 5′TGGCTGAATAAA**ACG**ACAGGAGTCTAT3′/5′ATAGACTCCTGT**CGT**TTTATTCAGC′.

### Transfections and luciferase assays

Jurkat cells (2 × 10^5^/well) were plated in 12 well plates and transfection was performed using Lipofectamine LTX as per manufacturer’s recommendation (Life Technologies, Grand Island, NY) using 1 μg pU3R-luc, 0.5 μg pCMV-Tax, and/or 2 μg MEF-2A, 1 μg shMEF-2A or 1 μg pHDAC9 plasmids. Where required, HEK 293 T cells were transfected with the indicated plasmids using GenJet according to the manufacturer’s instructions (SignaGen). In either case, cells were lysed 24 hr post-transfection using passive lysis buffer and luciferase activity was analyzed by the dual Luciferase Assay System (Promega, Madison, WI). Firefly luciferase values were normalized based on the *Renilla* luciferase internal control values. Each assay was performed in triplicate and repeated at least three times. A general estimate of transfection efficiency was obtained using the pMax-GFP (Lonza) plasmid (Life Technologies). Also, the shRNA-mediated inhibition of MEF-2A expression was confirmed in total RNA (Qiagen RNeasy kit) by RT-PCR with primers for MEF-2A forward/reverse 5′TCTCCACCTCAAACCACATTAC3′/5′CGTCCATCCTCATTCG CTTA3′, and GAPDH 5′GATTCCACCCATGGCAAATTC3′/5′GTCATGAGTCCTTCCA CGATAC3′. Tax expression upon pCMV-Tax transfection was also confirmed using Tax primers 5′CATGTACCTCTACCAGCTTT3′/5′GGGCAGGGCCCGGAAATCAT3′. In addition, extracellular LDH (Lactate Dehydrogenase) Cytotoxicity Assay (Pierce Thermo Scientific, Rockford, IL) to assess cellular viability post-transfection.

### Effect of MEF-2 inhibition on Tax expression, and HTLV-1 replication

Either scrambled or shMEF-2A plasmid (1 μg) was transfected into 10^7^ MT-2 cells using program U-014 on Amaxa Nucleofector II device (Lonza, Switzerland) following manufacturer’s recommendations. Cells were harvested at 24 and 48 hr and lysed in M-PER protein extraction reagent (Pierce). Lysates were sonicated and analyzed by Western blotting for MEF-2A (Cell Signaling Technologies, Danvers, MA), Tax (LT-4, 1:2000 dilution, provided by Dr. Yuetsu Tanaka, Japan) and β-actin (Millipore). Anti-MEF-2A Ab used here does not cross-react with related family members. To analyze effects of shMEF-2A on virus production, transfected MT-2 cells were washed at 48 hr and incubated in fresh medium for another 24–36 hr. Thereafter, supernatants were assessed for HTLV-1 core protein levels (pg/ml) by the p19-specific ELISA (ZeptoMetrix, Buffalo, NY).

Further, the transcriptional repressor MITR/HDAC9 was used to assess the influence of inhibiting MEF-2 activity on HTLV-1 replication. Briefly, 1 μg of this plasmid was transfected into 10^7^ MT-2 cells using Lipofectamine as above. Transfected cells were harvested every 24 hr over a 72 hr period and transcript analysis was performed by SYBR Green Cells-to-Ct procedure (Life Technologies) using the primer sets for Tax 5′CGTGTTTGGAGACTGTGTAC3′/5′CTG TTTCGCGGAAATGTTTT3′, p19 5′CACCCCTTTCCCTTTCATTCACGA3′/5′CCGGCCGG GGTATCCTTTT3′; and GAPDH 5′CAATGACCCCTTCATTGACC3′/5′TTGATTTTGGA GGGATCTGG3′. Quantitative PCR was performed using Applied Biosystems 7500 System, and fold-change in expression was calculated by 2^-ΔΔCT^ method [107].

### T-cell Transformation assay and Propidium Iodide (PI) staining

Peripheral blood mononuclear cells **(**PBMCs) were isolated using Ficoll-Paque (Amersham, Uppsala, Sweden) and stimulated with phytohemagglutinin (PHA 2 mg/ml) for 3 days as described [64]. Cells were transduced with lentivirus expressing either scrambled or shMEF-2A (Sigma) by spinoculation (1500 g for 3 hr) and incubated for 6 hr. Transduced cells were co-cultured with lethally irradiated (60 Gy) MT-2 cells at 1:5 ratio in presence of recombinant IL-2 (20 IU/ml, R&D Systems). After 3 weeks, puromycin (1 mg/ml) was added to media to select transduced cells and viable cells were counted at 3, 5 and 6 weeks.

For the cell cycle progression study, MT-2 and Jurkat cells were transfected with control or shMEF-2A plasmid as above and fixed in 70% ethanol overnight at −20°C. Cells were washed, stained (20 min, RT) with 300 μl of hypertonic buffer (PI-25 μg/ml, RNAase 40 μg/ml, sodium citrate-0.1%, and Triton-100 × −0.03%), and analyzed by flow cytometry. The resulting DNA distributions were analyzed by the Cell Proliferation tool in the software Flowjo (Tree Star Inc., USA), for the proportions of cells in G0-G1, S, and G2-M phases of the cell cycle.

### MEF-2 mRNA levels in clinical samples

PBMCs from three seronegative controls, asymptomatic carries and ATL patients were used from a Jamaican cohort, which was recently utilized in our studies [108,109]. These samples were obtained from National Cancer Institute, and processed under institutional review board guidelines. Total RNA from each sample was transcribed to cDNA and amplified in a standard real-time PCR reaction as described above using primers for MEF-2A (5ACCGAGAGGATAATTCAGTCCTG3′/5ACATCCGCGCAC GGATC3′), and GAPDH. RNA from Jurkat T cells was also amplified as control.

### Primary cells and infection

PBMCs were obtained as described above and CD4^+^ T cells were isolated by a negative selection kit (StemCell Technologies, Canada). Where indicated, labeled CD4^+^CD25^+^ T cells were isolated by positive selection for CD25^+^ from enriched CD4^+^ T cells. In each case, purity of cells was confirmed by flow cytometry. For infection, target cells (5 × 10^6^) were exposed to 125 ng/ml of HTLV-1 virus (Advanced Biotechnologies) for 72 hr. This preparation includes 4.62 × 10^11^ virus particles/ml in 1.2 mg/ml of total protein, which is equivalent to 0.5 mg/ml of HTLV-I Gag protein based on the p19 ELISA (ZeptoMetrix). We have successfully used this virus preparation in previous studies for different cell types [65,66]. Infection was verified by syncytia formation, Western blot analysis for Tax expression and intracellular Tax analysis by flow cytometry as per [65].

### Chromatin immunoprecipitation (ChIP) assay

Chromatin immunoprecipitation was performed using the ChIP-IT Express procedure as described by the manufacturer (Active Motif, Carlsbad, CA). Briefly 10^7^ cells were fixed with formaldehyde solution for 10 min and then washed with PBS; fixation was stopped using glycine-stop fix solution. The cells were lysed in a dounce homogenizer and sonicated for 10 pulses of 2 min each to obtain sheared DNA. The sheared chromatin was immunoprecipitated overnight at 4°C with 2 μg of the following antibodies: mouse monoclonal IgG (Santa Cruz Biotechnology, Santa Cruz, CA), CBP (Abcam, Cambridge, MA), pCREB (Santa Cruz Biotechnology), p300 (Abcam), p/CAF (Abcam), MEF-2 (Santa Cruz Biotechnology), Tax (LT-4) and p19 (ZeptoMetrix). Immunoprecipitated chromatin was eluted and then subjected to PCR using the following primer sets: LTR forward/reverse 5′GCCTCTCCTCCTACTTTTATGATG3′/5′ACCTTGGTCTCGTTTTCACT3′, and human GAPDH as above. PCR was performed in triplicate, and at least three independent samples were examined. Input DNA was used to draw a standard curve and to calculate % input as per the manufacturer’s instructions (Active Motif).

### Co-immunoprecipitation assay

First, protein levels of MEF-2A and other cellular factors were assessed in Jurkat, MT-2, control and HTLV-infected primary CD4^+^ T cells by Western blotting. Equal protein quantities from each sample were resolved by SDS-PAGE and transferred to a PVDF membrane. Membranes were blocked for 1 hr at room temperature with Odyssey blocking buffer (Li-Cor Biosciences) and then incubated with antibodies against β-actin (Millipore), p300, p/CAF, and CBP (Abcam), pCREB, and MEF-2A (SC-313, Santa Cruz), HDAC9 (Thermo-Pierce) for 1 hr. Membranes were incubated with IRDye-conjugated secondary antibodies and signals were detected using Odyssey Infrared Imager (Li-Cor). For Co-IP, cells were suspended in ice-cold lysis buffer [Tris–HCl pH 7.4 (25 mM), NaCl (150 mM), NP-40 (1%), EDTA (1 mM), glycerol (5%)] and sonicated. The supernatant was pre-cleared with protein A/G magnetic beads and immunoprecipitation with 2 μg of MEF-2A (Santa Cruz) or Tax (LT-4) antibody followed by Western blotting with same set of antibodies as above.

### Confocal microscopy

C8166 and MT-2 cells were cultured overnight on glass coverslips coated with poly-L-lysine. Cells were fixed with 1% paraformaldehyde and permeabilized with 0.2% Triton X-100. Fixed cells were incubated with SuperBlock buffer (Thermo Scientific) followed by staining with a Tax hybridoma (AIDS Research and Reference Program), and either anti-MEF-2A (Santa Cruz), anti-CREB (48H2; Cell Signaling), or anti-IRAK1 (D51G7; Cell Signaling) rabbit antibodies. Cover slips were incubated with Alexa Fluor 555-conjugated donkey anti-mouse IgG, Alexa Fluor 488-conjugated donkey anti-rabbit IgG (Life Technologies) and DAPI. Images were obtained using a Nikon C1si confocal microscope.

### Effects of MEF-2A inhibition on transcriptional factors recruitment to the LTR

The ability of MEF-2 and other cellular TFs to physically interact with the HTLV-1 LTR was investigated using a Promoter-Binding TF Profiling Array (Signosis, Sunnyvale, CA), in the absence or presence of shMEF-2A, as above. This is a competition assay in which nuclear extract is incubated with 48 different Biotin-labeled oligonucleotide probes each having affinity to a single TF. After incubation, TF-bound complexes are eluted, denatured and hybridized in a 96-well plate where each well has a complementary DNA of a specific probe to capture it. After capture, oligos are detected using streptavidin-HRP and a chemiluminescent substrate. Assay was performed as recommended by the manufacturer (Signosis). Briefly, nuclear extract was isolated from 10^6^ MT-2 cells and the protein content was estimated by Bradford assay. The reaction mixture was prepared using 15 μl TF binding buffer, 3 μl probe, 10 μg nuclear extract and 5 μl of HTLV-1 LTR or a control DNA, and incubated at room temperature for 30 min to allow formation of the TF-DNA complex. Unbound probes were separated from the complex, while bound probes were eluted and then hybridized to the plate and incubated overnight at 42°C. Bound probe was detected using HRP-streptavidin conjugate incubated with the chemiluminiscent substrate. Chemiluminiscence was measured using TopCount NXT luminescence counter (PerkinElmer).

This assay was also performed by knocking down Tax in MT-2 cells by the siRNA approach (Sense strand: 5′ rGrGrC rCrUrU rArUrU rUrGrG rArCrA rUrUrU rAdTdT 3′; Antisense strand: 5′ rUrArA rArUrG rUrCrC rArArA rUrArA rGrGrC rCdTdT 3′, IDT) using Lipofectamine RNAiMAX transfection reagent. (Life Technologies). Both control and siTax-transfected cells were subjected to the promoter binding assay as well as a Calcineurin Cellular Activity Assay (Enzo Life Sciences), for which 5 μg of total protein was incubated with the RII phosphopeptide (a calcineurin substrate) for 30 min. Following incubation, free-phosphate released was detected colorimetrically (OD at 620 nm) after adding BIOMOL GREEN™ reagent (based on the classic Malachite green assay). Each condition was performed in duplicate and the human recombinant calcineurin was included as a positive control.

### Electrophoretic mobility shift assay (EMSA)

Small-scale nuclear extracts were prepared from cells as described previously [110]. The following sequences were used to generate double-stranded oligonucleotides for EMSA: MEF-2 site from HTLV-1 LTR: 5′GAATAAA**CTAACAGGAG**TCT3′ (biotinylated at 5′ end), MEF-2 consensus site from Glut4 promoter [111]: 5′GGGAG**CTAAAAATAG**CAG3′, MEF-2 consensus mutant: 5′GGGAG***ACG*AAAA*CCG***CAG3′ and Oct-1: 5′TGTCGAATGCAAATCACTAGAA3′ (biotinylated at 5′ end). Nonradioactive EMSA was performed using LightShift Chemiluminescent EMSA Kit (Thermo Scientific) according to the manufacturer’s instructions. Western blotting was performed essentially as described above. Whole-cell lysates were resolved by SDS-PAGE, transferred to nitrocellulose membranes, blocked in 5% milk, incubated with the indicated primary and secondary antibodies, and detected using Western Lightning enhanced chemiluminescence reagent (Perkin Elmer). Anti-FLAG M2 was purchased from Sigma. Anti-β-actin (AC15) was from Abcam.

### Protein/DNA array to profile MEF-2 integrating signaling pathways upon HTLV-1 infection of primary CD4^+^ T cells

The activation status of various cellular factors was assessed by utilizing a Protein-DNA Array I (Panomics) exactly as previously published [69]. Briefly, nuclear extract was isolated from 10^6^ cells and mixed with biotinylated probes to allow the formation of protein-DNA complexes. Labeled probes were eluted from free probes, hybridized to the pretreated array membranes and scanned using the FluorChem Imager (Alpha Innotech). Spots were quantified by Image J software and normalized to their respective controls after background subtraction. The key identified factors from arrays were verified by Western blotting for phosphorylated p38 MAPK (Life Technologies), phospho-ERK5, phospho-Smad2, phospho-Akt, and phophoMEF-2A (Cell Signaling).

## Additional files

Additional file 1: Figure S1. (A) Transfection efficiency of Jurkat cells determined with plasmid pMax-GFP (Lonza) transfected with Lipofectamine LTX in triplicate as described in Methods. Transfected cells were collected 24 hr post-transfection and analyzed by flow cytometry. Number represents the transfection efficiency in as percent of GFP-positive cells. (B) Inhibition of MEF-2 mRNA expression by shRNA was determined by RT-PCR. RNA isolated from transfected Jurkat cells was converted to cDNA and then amplified using MEF-2A and Tax primers described in Methods. The PCR product was then run using a DNA gel and presence of MEF-2A and Tax was confirmed. (C) LDH cytotoxicity assay was performed on transfected cells to measure extracellular LDH in culture media. Spectrophotmetric measurement of a red formazan product was used to confirm that transfection did not affect cellular viability. (D) HTLV-1 LTR luciferase assays in 293 T cells transfected with empty vector (Mock) or LTR luciferase plasmid (LTR Luc) without or with plasmids for Tax, shMEF-2A, HDAC9, and MEF-2A. Luciferase values are presented as “fold induction” relative to the control (EV). Two-tailed unpaired *t-test* was performed with Prism software. Error bars represent the standard deviation of triplicate samples. The level of significance was defined as *p < 0.05, **p < 0.01. NS = not significant. Additional file 2: Figure S2. The infection of primary CD4^+^ T cells and cell lines was verified by confirmation of Tax expression. Tax expression was confirmed in cell lines and primary cells using (A) Flow cytometry, and (B) Western Blot. Tax mAb (clone LT-4) was used for both flow cytometry and Western blot. For flow cytometry, allophycocyanin (APC) was conjugated on Tax mAb. Additional file 3: Figure S3. HTLV-1 infection does not change GAPDH expression. Amplification plots are shown following qPCR analysis for GAPDH expression in cell lines and primary cells. Additional file 4: Figure S4. Core protein p19 is not promoter bound during HTLV-1 infection. Quantitative PCR amplification plots following ChIP analysis show that the viral core protein is not recruited to cellular or viral promoters. Additional file 5: Figure S5. Validation of MEF-2 interaction with Tax in the alternate cell systems. (A) Immunoblotting was performed after IgG control or Tax immunoprecipitation with the indicated antibodies using whole cell lysates derived from C8166 cells. This data confirms that Tax interacts with MEF2 in C8166 cells. (B) Mock or FLAG-Tax expression plasmid was transfected in 293 T cells for 36 hr. Expression of Tax was confirmed in the lysate and it was precipitated using an anti-FLAG antibody followed by immunoblotting with the anti-MEF-2 antibody. Additional file 6: Figure S6. Inhibition of Tax impacts calcineurin activity in MT-2 cells. One million MT-2 cells were either mock transfected (Control) or transfected with siRNA against Tax using Lipofectamine RNAiMAX Transfection Reagent (Life Technologies) according to manufacturer’s protocol. After 72 hr, cell lysate was prepared using M-PER (Pierce) and then used to measure calcineurin activity using Calcineurin Cellular Activity Assay kit (Enzo Life Sciences). Briefly, 5 μg total protein (from control as well as siTax transfected cells) was incubated with the RII phosphopeptide (a calcineurin substrate) for 30 min. Following incubation, free-phosphate released was detected colorimetrically (OD at 620 nm) after adding BIOMOL GREEN™ reagent (based on classic Malachite green assay). Human recombinant calcineurin was included as a positive control. Two replicates were used and p-value (0.047) was calculated using Student’s *t*-test (One-tailed). Additional file 7: Table S1. Raw and average intensities of analyzed cellular factors in the promoter binding assay performed with MT-2 cells. Additional file 8: Figure S7. The transcriptome is upregulated and multiple signaling pathways are activated upon HTLV-1 infection. Schematic representation of Promoter-Binding Transcription Factor Profiling Assay (Signosis) (A). Protein-DNA Array plate hybridized with labeled probes against selected transcription factors each representing a canonical signaling pathway. Chemiluminescent images collected using the FluorChem™ Imager (Alpha Innotech) reveal changes in protein binding activities of the transcription factors indicating activation of multiple signaling pathways upon HTLV-1 infection (B).
