# Supplementary figures and images for: Myocyte enhancer factor (MEF)-2 plays essential roles in T-cell transformation associated with HTLV-1 infection by stabilizing complex between Tax and CREB
Source: Retrovirology. 2015 Feb 27;12:23. doi: 10.1186/s12977-015-0140-1 (PMC4374383; doi:10.1186/s12977-015-0140-1)

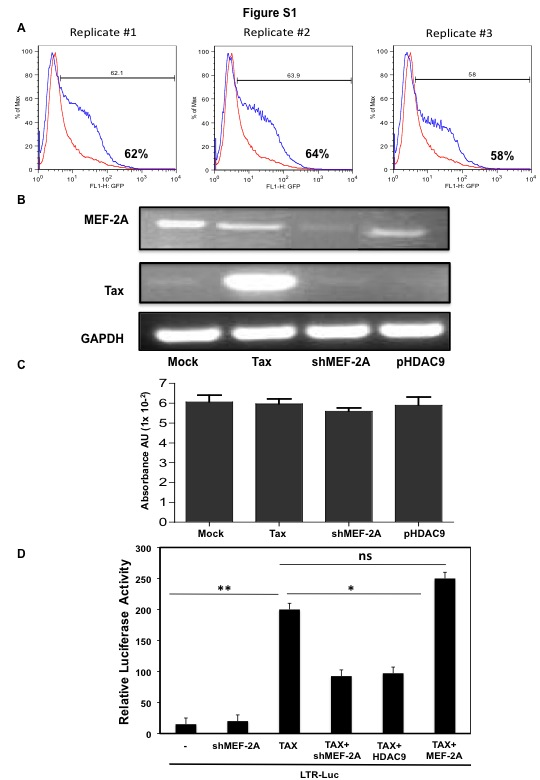

Supplement: Additional file 1: Figure S1. — (A) Transfection efficiency of Jurkat cells determined with plasmid pMax-GFP (Lonza) transfected with Lipofectamine LTX in triplicate as described in Methods. Transfected cells were collected 24 hr post-transfection and analyzed by flow cytometry. Number represents the transfection efficiency in as percent of GFP-positive cells. (B) Inhibition of MEF-2 mRNA expression by shRNA was determined by RT-PCR. RNA isolated from transfected Jurkat cells was converted to cDNA and then amplified using MEF-2A and Tax primers described in Methods. The PCR product was then run using a DNA gel and presence of MEF-2A and Tax was confirmed. (C) LDH cytotoxicity assay was performed on transfected cells to measure extracellular LDH in culture media. Spectrophotmetric measurement of a red formazan product was used to confirm that transfection did not affect cellular viability. (D) HTLV-1 LTR luciferase assays in 293 T cells transfected with empty vector (Mock) or LTR luciferase plasmid (LTR Luc) without or with plasmids for Tax, shMEF-2A, HDAC9, and MEF-2A. Luciferase values are presented as “fold induction” relative to the control (EV). Two-tailed unpaired t-test was performed with Prism software. Error bars represent the standard deviation of triplicate samples. The level of significance was defined as *p < 0.05, **p < 0.01. NS = not significant. [file 12977_2015_140_MOESM1_ESM.tiff]

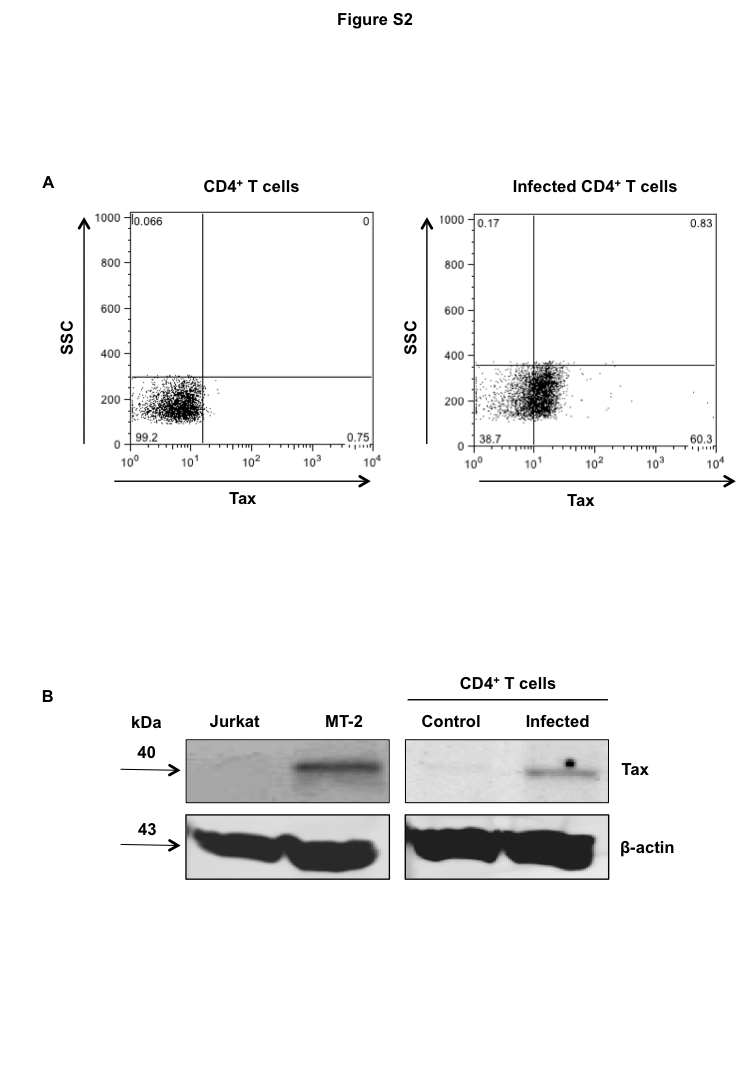

Supplement: Additional file 2: Figure S2. — The infection of primary CD4+ T cells and cell lines was verified by confirmation of Tax expression. Tax expression was confirmed in cell lines and primary cells using (A) Flow cytometry, and (B) Western Blot. Tax mAb (clone LT-4) was used for both flow cytometry and Western blot. For flow cytometry, allophycocyanin (APC) was conjugated on Tax mAb. [file 12977_2015_140_MOESM2_ESM.tiff]

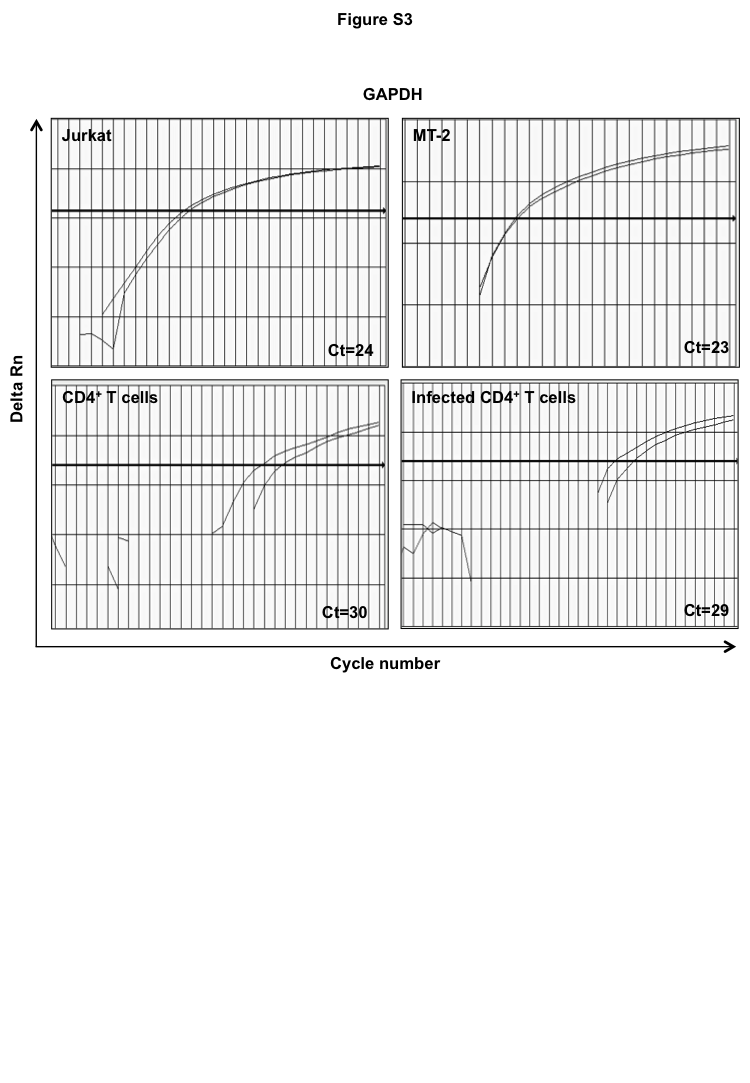

Supplement: Additional file 3: Figure S3. — HTLV-1 infection does not change GAPDH expression. Amplification plots are shown following qPCR analysis for GAPDH expression in cell lines and primary cells. [file 12977_2015_140_MOESM3_ESM.tiff]

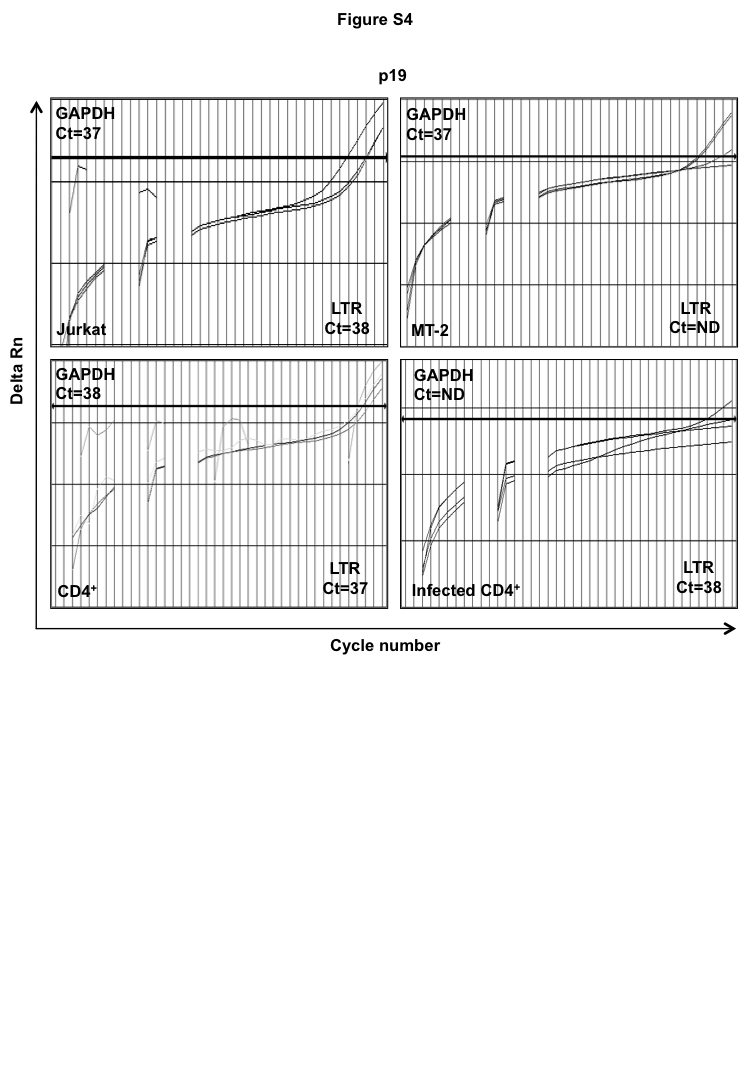

Supplement: Additional file 4: Figure S4. — Core protein p19 is not promoter bound during HTLV-1 infection. Quantitative PCR amplification plots following ChIP analysis show that the viral core protein is not recruited to cellular or viral promoters. [file 12977_2015_140_MOESM4_ESM.tiff]

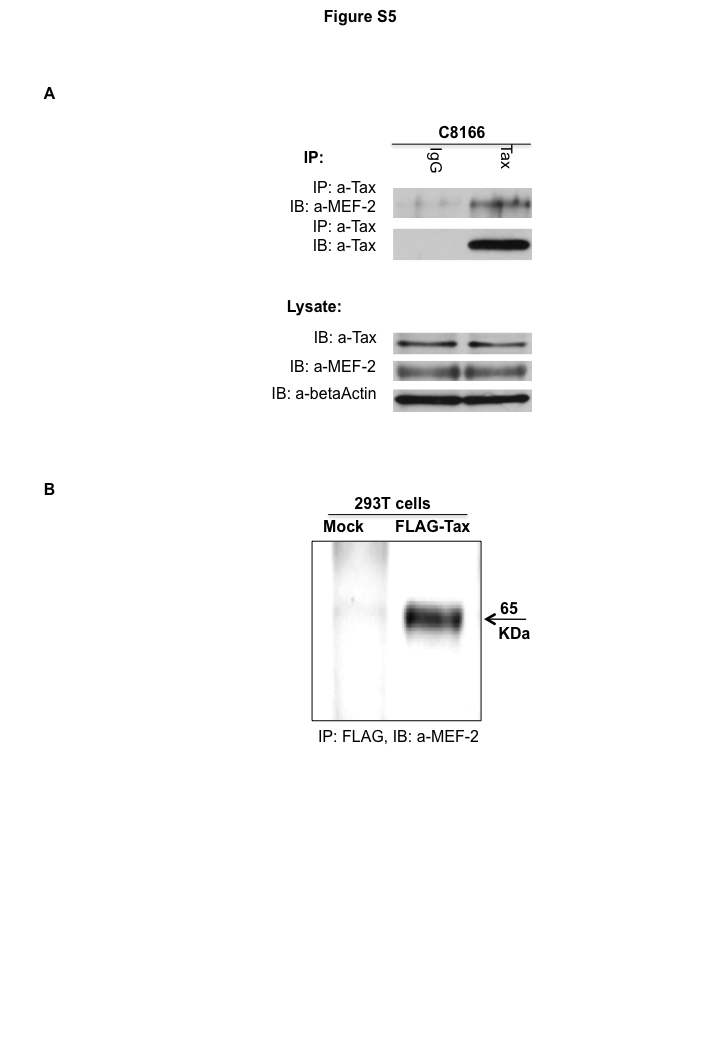

Supplement: Additional file 5: Figure S5. — Validation of MEF-2 interaction with Tax in the alternate cell systems. (A) Immunoblotting was performed after IgG control or Tax immunoprecipitation with the indicated antibodies using whole cell lysates derived from C8166 cells. This data confirms that Tax interacts with MEF2 in C8166 cells. (B) Mock or FLAG-Tax expression plasmid was transfected in 293 T cells for 36 hr. Expression of Tax was confirmed in the lysate and it was precipitated using an anti-FLAG antibody followed by immunoblotting with the anti-MEF-2 antibody. [file 12977_2015_140_MOESM5_ESM.tiff]

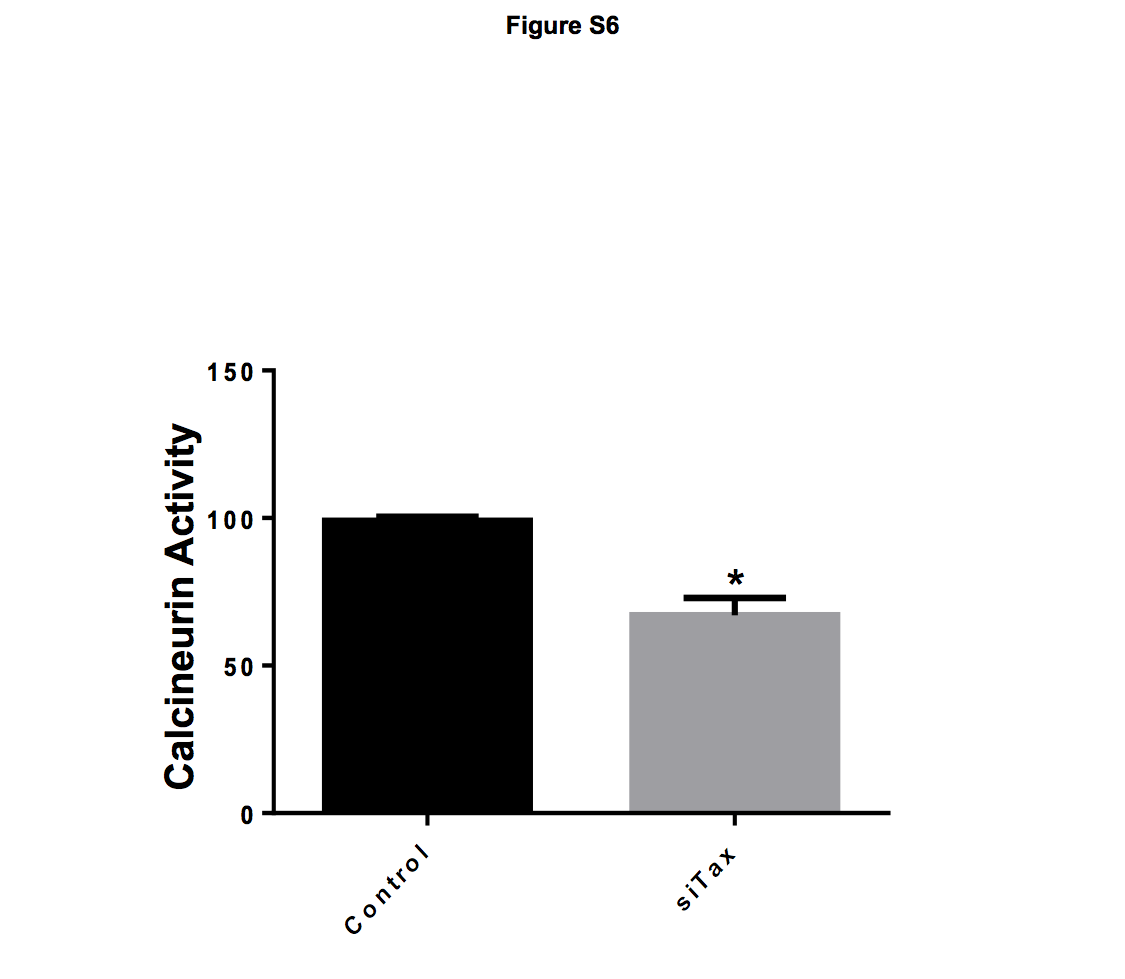

Supplement: Additional file 6: Figure S6. — Inhibition of Tax impacts calcineurin activity in MT-2 cells. One million MT-2 cells were either mock transfected (Control) or transfected with siRNA against Tax using Lipofectamine RNAiMAX Transfection Reagent (Life Technologies) according to manufacturer’s protocol. After 72 hr, cell lysate was prepared using M-PER (Pierce) and then used to measure calcineurin activity using Calcineurin Cellular Activity Assay kit (Enzo Life Sciences). Briefly, 5 μg total protein (from control as well as siTax transfected cells) was incubated with the RII phosphopeptide (a calcineurin substrate) for 30 min. Following incubation, free-phosphate released was detected colorimetrically (OD at 620 nm) after adding BIOMOL GREEN™ reagent (based on classic Malachite green assay). Human recombinant calcineurin was included as a positive control. Two replicates were used and p-value (0.047) was calculated using Student’s t-test (One-tailed). [file 12977_2015_140_MOESM6_ESM.tiff]

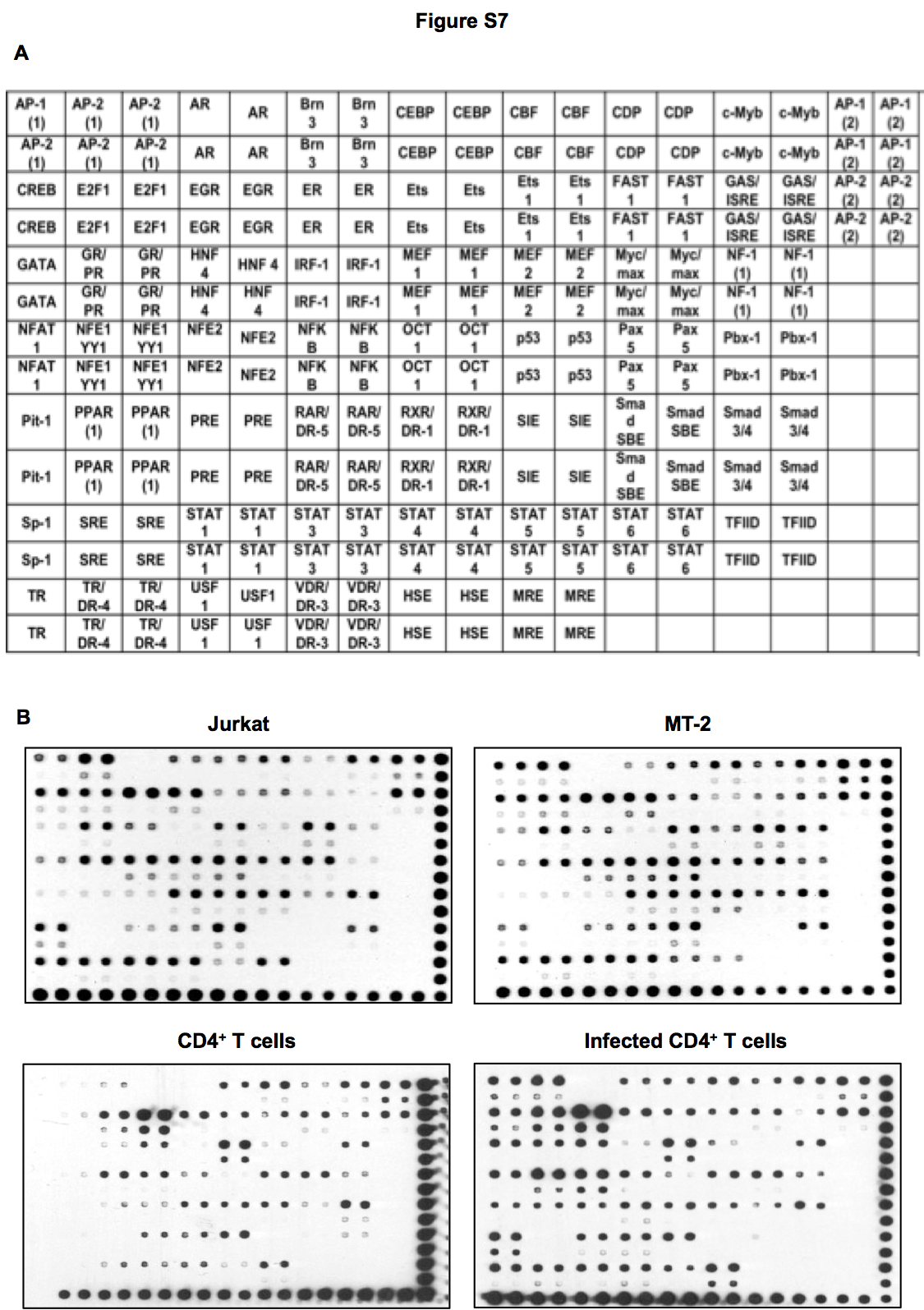

Supplement: Additional file 8: Figure S7. — The transcriptome is upregulated and multiple signaling pathways are activated upon HTLV-1 infection. Schematic representation of Promoter-Binding Transcription Factor Profiling Assay (Signosis) (A). Protein-DNA Array plate hybridized with labeled probes against selected transcription factors each representing a canonical signaling pathway. Chemiluminescent images collected using the FluorChem™ Imager (Alpha Innotech) reveal changes in protein binding activities of the transcription factors indicating activation of multiple signaling pathways upon HTLV-1 infection (B). [file 12977_2015_140_MOESM8_ESM.tiff]
